# Genomic, transcriptomic, and phenotypic differences among archetype *Shigella flexneri* strains of serotypes 2a, 3a, and 6

**DOI:** 10.1128/msphere.00408-23

**Published:** 2023-10-13

**Authors:** Caitlin E. Gabor, Tracy H. Hazen, BreOnna C. Delaine-Elias, David A. Rasko, Eileen M. Barry

**Affiliations:** 1 Institute for Genome Sciences, University of Maryland School of Medicine, Baltimore, Maryland, USA; 2 Department of Microbiology and Immunology, University of Maryland School of Medicine, Baltimore, Maryland, USA; 3 Center for Vaccine Development and Global Health, University of Maryland School of Medicine, Baltimore, Maryland, USA; 4 Center for Pathogen Research, University of Maryland School of Medicine, Baltimore, Maryland, USA; University of Michigan-Ann Arbor, Ann Arbor, Michigan, USA

**Keywords:** *Shigella*, *Shigella flexneri*, shigellosis, comparative genomics

## Abstract

*Shigella* is one of the leading causes of diarrheal disease in low-to-middle income countries, where it accounts for significant disease burden in children under the age of five. *Shigella flexneri*, one of four *Shigella* species, is composed of 15 serotypes and caused 65.9% of all shigellosis cases within the Global Enteric Multicenter Study. *S. flexneri* serotypes 2a, 3a, and 6 are the three leading serotypes implicated in clinical disease, yet there are limited studies examining the variation of pathogenesis and virulence between serotypes. Our data revealed through *in silico* and *in vitro* analysis that several serotype-pecific differences exist between archetype strains 2457T (*S. flexneri* serotype 2a [*Sf*2a]), J17B (*S. flexneri* serotype 3a [*Sf*3a), and CCH060 (*S. flexneri* serotype 6 [*Sf6*]). Comparative genomics of these archetype strains demonstrated that CCH060 contains the greatest amount of unique genomic content compared to the *Sf*3a and *Sf*2a archetype strains, as well as lacks several previously identified *Shigella* virulence genes. The pINV virulence plasmid contains a highly conserved core genome irrespective of the archetype reference strain with the greatest strain-specific genomic features. The transcriptional responses of each archetype strain to bile salt stimulus were unique in a strain-dependent manner. Phenotypic analysis revealed that while archetype strains adhere at similar levels to cultured HT-29 intestinal cells, there are differences in the production and secretion of IpaB, IpaC, and IpaD, as well as reduced invasion by CCH060 compared to 2457T and J17B. Our results identify strain-specific features that support further large-scale analysis to identify unique serotype-specific host-pathogen interactions.

**IMPORTANCE:**

Given the genomic diversity between *S. flexneri* serotypes and the paucity of data to support serotype-specific phenotypic differences, we applied *in silico* and *in vitro* functional analyses of archetype strains of 2457T (*Sf*2a), J17B (*Sf*3a), and CH060 (*Sf*6). These archetype strains represent the three leading *S. flexneri* serotypes recommended for inclusion in multivalent vaccines. Characterizing the genomic and phenotypic variation among these clinically prevalent serotypes is an important step toward understanding serotype-specific host-pathogen interactions to optimize the efficacy of multivalent vaccines and therapeutics. This study underpins the importance for further large-scale serotype-targeted analyses.

## INTRODUCTION

*Shigella* spp. are a causative agent of moderate-to-severe diarrhea and are responsible for ≥200,000 deaths per year (1). In the Global Enteric Multicenter Study (GEMS), a 3-year case-control study of diarrhea in children under the age of 5 in low-to-middle income countries (LMICs), *Shigella* was identified as one of four leading pathogens in all age groups and the top pathogen in children between 1 and 5 years of age (2, 3). Despite significant public health efforts, *Shigella* continues to be a major concern of residents, travelers, and military personnel deploying to endemic regions (4–7). Antimicrobial compounds are one of the primary treatments; however, emerging resistance among *Shigella* to common antibiotics such as fluroquinolones highlights the need for alternative treatment options (8).

Several promising vaccine candidates have entered clinical development over the past decade, including serotype-based vaccines, conserved antigen vaccines, and combinations of the two (9–11). To date, vaccine development has been hindered largely by the number of serotypes and genomic diversity of *Shigella* (12, 13); a *Shigella* vaccine has yet to be licensed for human use. *Shigella* is composed of four distinct species: *Shigella dysenteriae*, *Shigella flexneri*, *Shigella boydii*, and *S. sonnei* (13, 14). Each *Shigella* species, apart from *S. sonnei*, is composed of multiple serotypes (7). *S. flexneri* has 15 serotypes; each serotype has a unique O-antigen composition which can be identified using antisera or monoclonal antibodies (15, 16), or defined and grouped by genomic content (17). *S. flexneri* is the predominant *Shigella* species causing pediatric shigellosis in LMICs (3, 18, 19) and accounted for 65.9% of shigellosis cases in GEMS (20). Of the 837 samples containing *S. flexneri*, *S. flexneri* serotype 2a (*Sf*2a) (*n* = 245, 29.3%), *S. flexneri* serotype 3a (*Sf*3a) (*n* = 123, 14.7%), and *S. flexneri* serotype 6 (*Sf*6) (*n* = 156, 18.6%) were the three most prevalent (3, 21).

Phylogenomic distribution of *S. flexneri* reveals two distinct lineages: one compromised of *S. flexneri* serotypes 1–5, 7, and X-Y, which can be further delineated into seven phylogenetic groups (PGs 1–7), as well as a distant lineage composed only of *Sf*6 (17). Despite these phylogenomic differences, *Sf*6 contributes significantly to the disease burden across geographic regions within Africa, Asia, and Latin America (9, 12). To date, *Sf*6 is has been poorly characterized genomically and phenotypically and rarely compared to the other serotypes (22).

Irrespective of the *S. flexneri* serotype, following ingestion of contaminated food or water, *Shigella* spp. traverse the gastrointestinal tract to the small intestine, where they are exposed to bile salts, organic components of bile produced by the liver and stored in the gall bladder (23). Bile salts have detergent-like properties that confer antimicrobial activity by compromising the bacterial membrane integrity (24). However, some enteric pathogens, including *Shigella* spp., can resist bile salt-mediated death and exploit this host component as a signal to modulate virulence gene expression and enhance infection (24–26). At the ileal and colonic mucosal surfaces, *Shigella* penetrates through the mucus layer to reach the epithelial cell surface and triggers transcytosis via microfold (M) cells (27–29). *Shigella* are then phagocytosed by resident macrophages residing in the basal pocket of M cells. The bacteria escape the phagocytic vacuole, induce pyroptosis, and escape macrophages to invade epithelial cells via the basolateral surface by triggering actin cytoskeleton reorganization through the secretion of virulence effectors via the type III secretion system (T3SS) (30–33). The genes required for bacterial entry into epithelial cells are present on a 30-kb region of the pINV plasmid (34). The entry region contains the Mxi-Spa genes responsible for the T3SS scaffold, secreted Ipa A-D virulence effectors, Ipg proteins and chaperones, and the VirB transcription regulator (35). The Ipa proteins are critical effectors that play a role in pore formation, vacuolar escape, and actin cytoskeleton remodeling involved in invasion (36, 37). Once inside the epithelial cells, *Shigella* spp. escape the vacuole, multiply intracellularly, and spread cell-to-cell (38, 39). Within the epithelium, *Shigella* invasion triggers production and secretion of interlukin-8 (IL-8), which recruits neutrophils and promotes invasion of more *Shigella* (40, 41). The resulting inflammatory response disrupts the epithelium, leading to bloody, mucoid diarrhea (42).

The paucity of data and literature to support serotype-specific phenotypic differences of *Shigella* prompted our studies to apply *in silico* and *in vitro* functional analysis of archetype strains of *Sf*2a, *Sf*3a, and *Sf*6 (2, 13, 17, 22, 43–45). The archetype strains included in this study, 2457T (*Sf2a*), J17B (*Sf3a*), and CCH060 (*Sf6*), are historical clinical strains that have been extensively utilized in previous *S. flexneri* functional studies (45–48). These archetype strains represent the three leading *S. flexneri* serotypes recommended for inclusion in multivalent vaccines (2). Characterizing the genomic and phenotypic variation among these clinically prevalent serotypes is an important step toward understanding serotype-specific host-pathogen interactions to optimize the efficacy of multivalent vaccines and therapeutics.

## RESULTS

### Generation of whole-genome sequences for the archetype strains

Whole-genome sequencing was used to examine *S. flexneri* archetype strains 2457T, J17B, and CCH060 (Table 1). *Sf*2a strain 2457T assembled into a single circularized chromosome of ~4.65 Mb in addition to four plasmids. The plasmids included three plasmids with sizes ranging from ~9 to 165 kb, in addition to the ~221-kb IncFII plasmid p2457T_221 that has been previously characterized as the *S. flexneri* virulence plasmid, pINV (49). The 221-kb plasmid contains genes for the *mxi*/*spa* and *ipa* loci, which encode 20 proteins that produce the extracellular needle and tip complex of the T3SS, as well as secreted effectors required for *Shigella* virulence (36, 49, 50). Annotation revealed p2457T_164, the second largest plasmid, which was previously identified in 2457T (51), contained several genes encoding hypothetical proteins (58.5%, 113 of 193) and no established *Shigella* virulence markers or antimicrobial resistance genes (Data Set 1). Two small cryptic plasmids, p2457T_9 and p2457T_8, were identified in strain 2457T that had not been sequenced previously (51). Of note, previous genomic sequencing of *S. flexneri* 2457T included only the chromosome (51), whereas the genome in this study is the complete genomic content from a single culture. *Sf*3a strain, J17B, assembled into a single circularized chromosome of ~4.7 Mb and the single, un-circularized IncFII virulence plasmid of 264.7 kb (Table 1). *Sf*6 strain, CCH060, assembled into a single, un-circularized chromosome of ~4.68 Mb and two plasmids including a ~195.8-kb IncFII virulence plasmid and a ~77-kb Inc1 plasmid (Table 1). The role of pCCH060_77 in CCH060 is unknown; it contains several genes encoding hypothetical proteins (20.5%, 17 of 83), in addition to several phage components and type II/type VI secretion system family proteins (Table 1, Data Set 1).

**TABLE 1:** Characteristics of the complete *S. flexneri* genome assemblies

Isolate characteristics						Assembly characteristics					
Isolate	Serotype	Phylogenetic group^*a*^	No. of contigs	Genome size (Mb)	Overall GC %	Contig label	Contig description	Sequence length (bp)	GC %	Plasmid incompatibility types	GenBank accession no.
2457T	2a	PG3	5	5.05	50.76	2457T_1	Chromosome	4,643,553	50.92	None	CP100044
						p2457T_221	Virulence plasmid	221,974	45.86	IncFII(AY458016)	CP100045
						p2457T_165	Plasmid	165,691	45.70	IncFIA(HI1), IncHI1A, IncHI1B(R27)	CP100046
						p2457T_9	Plasmid	9,532	45.06	None	CP100047
						p2457T_8	Plasmid	8,219	52.79	None	CP100048
J17B	3a	PG4	2	4.97	50.73	J17B_1	Chromosome	4,709,683	50.98	None	CP100042
						pJ17B_264	Virulence plasmid	264,790	46.22	IncFII(AY458016)	CP100043
CCH060	6	None	3	4.96	50.97	CCH060_1	Chromosome	4,684,126	51.21	None	CP099865
						pCCH060_195	Virulence plasmid	195,804	45.40	IncFII(AY458016)	CP099866
						pCCH060_77	Plasmid	77,769	50.24	IncI1Alpha(AP005147)	CP099867

^*a*^ As indicated by previous study (17).

### Genomic variation of *S. flexneri* strains 2457T, J17B, and CCH060

**FIG 1:**
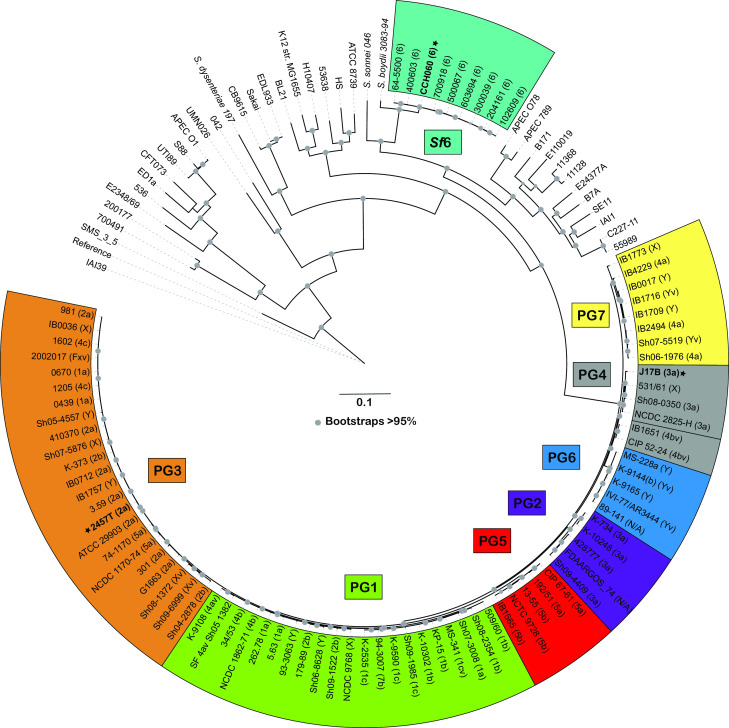
Phylogenomic analysis of the archetype strains for *S. flexneri* serotype 2a, 3a, and 6. The representative *Sf*2a (2457T), *Sf*3a (J17B), and *Sf*6 (CCH060) genomes analyzed in this study were compared with 79 previously sequenced genomes representing diverse *S. flexneri* phylogenomic groups and serotypes and a collection of 37 diverse *E. coli* and *Shigella* genomes (Table S1). The phylogenomic groups are designated PG1 to PG7, as described in a previous study (17), and the serotypes where known are indicated in parentheses next to each strain name. The archetype strain genomes sequenced in this study are indicated in bold with a star.

A whole-genome phylogeny was generated comparing the genomes of the archetype strains for 2457T (*Sf*2a), J17B (*Sf*3a), and CCH060 (*Sf*6) to 79 previously sequenced *S. flexneri* genomes and 37 diverse reference *Escherichia coli* and *Shigella* genomes (Table S1). The inferred phylogeny illustrates the diversity of the sequenced archetype strains in the context of an array of *Shigella* spp. and *E. coli* strains (Fig. 1). *S. flexneri* strains 2457T, J17B, and CCH060 group within the previously characterized *S. flexneri* genomes but separate from the representative *E. coli* genomes (17). The archetype strains 2457T and J17B are within the genomic lineage that contains most of the *S. flexneri* serogroups, which can be subdivided into PGs based on their genomic content, as previously described (17). *Sf*2a strain 2457T was identified in PG3 with all other representative serotype 2a and some 2b genotypes analyzed. *Sf*3a strain J17B was identified in PG4, one of two PGs that contain *Sf*3a strains (17).

In contrast, *Sf*6 strains group together in a separate, independent lineage from the other *S. flenxeri* serotypes as previously noted (12). *Sf*6 strains, including archetype strain CCH060, publicly available strain 64-5500, as well as representative clinical strains, do not group within any of the PGs (17). As previously identified, *Sf*6 strains group with *S. boydii* 3083-94 (*S. boydii* serotype 4) (13). Overall, the phylogenetic analysis highlights the distinct genomic content of *Sf*6 strain CCH060 compared to the other *S. flexneri* serotype strains.

### Comparison of the gene content of *S. flexneri* archetype strains 2457T, J17B, and CCH060

**FIG 2:**
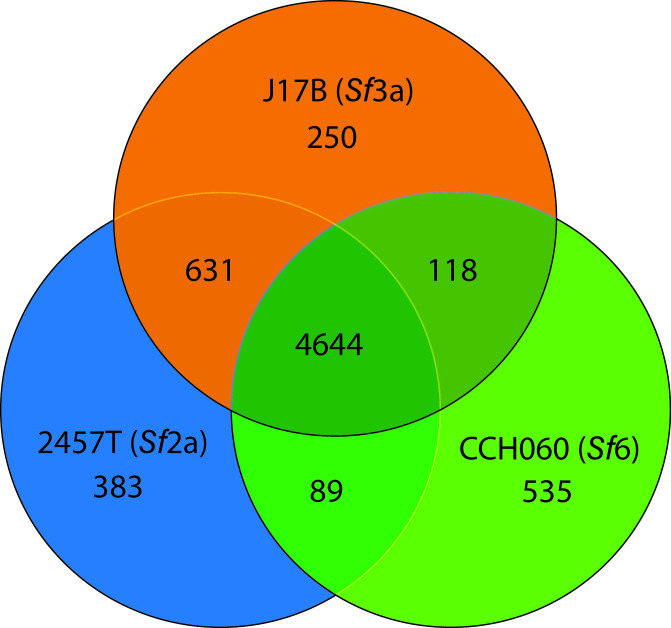
Whole-genome comparisons of 2457T, J17B, and CCH060. Archetype strains 2457T (blue), J17B (orange), and CCH060 (green) genomes were analyzed using *de novo* LS-BSR to compare the genomes to each other in an unbiased manner (52). A BSR value of ≥0.80 was used to identify predicted genes that were present, and a BSR value of ≤0.40 was used to identify predicted genes that were absent. Based on BSR scores, predicted genes were separated into core (shared between three), shared (shared between two), and accessory or variable (unique) genomic features (Data Set 2).

To identify gene content that was differentially distributed between the three archetype strains, a large-scale blast score ratio (LS-BSR) analysis was used (52). A blast score ratio (BSR) of ≥0.80 identified predicted genes present and ≤0.40 identified predicted genes that were absent from each strain. The three archetype strains shared 4,644 conserved genes (95%; 4,415 chromosomes, 4.9%; 229 plasmid), including established virulence genes such as the *mxi/spa loci*, *ipaB*, *ipaC*, *ipaD*, *ospD3* (ShET2 enterotoxin), as well as multidrug resistance protein D (Fig. 2, Data Set 2). The distribution of the accessory genomic content was more pronounced for CCH060, reflecting its phylogenomic distance from 2457T and J17B (Fig. 1). Strains 2457T and J17B shared 631 genes that are absent from CCH060, including predicted virulence genes *ospG*, *sepA*, *emrE* (multidrug transport system), and *matE* (multi-antimicrobial exclusion protein), as well as genes involved in various metabolic processes (Data Set 2).

A total of 383 unique predicted genes are identified in 2457T (Fig. 2). Of these unique genes, 43.1% (165 of 383) were annotated as hypothetical proteins. Strain 2457T’s three unique plasmids (p2457T_165, p2457T_9, and p2457T_8; Table 1) are composed primarily of hypothetical proteins whose function is undetermined (Data Sets 1 and 2). The *set1A* and *set1B* genes, which encode ShET1 enterotoxin, unique to *Sf*2a and some *Sf*2b strains (53), are uniquely present within strain 2457T (Data Set 2). In *Sf*3a strain J17B, there are 250 unique predicted genes (Fig. 2). Of these, 31.2% (78 of 250) are annotated as hypothetical proteins. Predicted genes of interest include unique phage components and biofilm export pores (*pgaABCD*) (Data Set 2). The greatest number of predicted unique genes, 535, was identified in *Sf*6 strain CCH060 (Fig. 2). Of those unique genes, 24.7% (132 of 535) were annotated as hypothetical proteins. Predicted genes of interest include unique phage components, penicillin amidase family proteins, a CRSPR/cas system, and type II secretion system (T2SS) components (Data Set 2).

Our unbiased analysis indicated genomic variation in the presence of virulence genes between archetype strains (Data Set 2). An in-depth analysis into the presence of a set of characterized *Shigella* virulence genes (54) is shown in Table 2. *Sf*2a strain 2457T contains all the characterized *Shigella* virulence genes evaluated in this analysis (Table 2). All archetype strains contained the *mxi*/*spa*/*ipa* genes, which are essential in virulence (54), as well as *ospD3* (ShET2 enterotoxin), *virG*, and *virF* (Table 2). *Sf*6 strain CCH060 contained the fewest virulence genes of the three archetype strains. CCH060 is lacking plasmid virulence genes *sepA* and *ospG*, and chromosomal encoded virulence genes *pic* and *shiA,* along with the SHI-O pathogenicity island implicated in O-antigen modification (Table 2). Endpoint PCR was used to confirm the absence of these genes from CCH060 (Table S4).

**TABLE 2:** LS-BSR *Shigella* virulence genes^*a*^

		Strain/serotype		
Gene(s) or PAI^*d*^	Chromosome or plasmid	2457T (*Sf*2a)	CCH060 (*Sf*6)	J17B (*Sf*3a)
*mxi/spa/ipa*	Plasmid	+	+	+
ShET1 (*set1A*, *set1B*)	Chromosome	+	−	−
ShET2 (*sen*)	Plasmid	+	+	+
SHI-1				
*pic^b^*	Chromosome	+	−	−
*sigA^b^*	Chromosome	+	+	−
SHI-2				
*iucA-D*	Chromosome	+	+	+
*iutA^c^*	Chromosome	+	−	+
*shiA^b^*	Chromosome	+	−	+
*shiB-E*	Chromosome	+	+	+
SHI-O				
*gtrA, B*	Chromosome	+	−	+
*gtrII^b^*	Cromosome	+	−	−
*sepA^b^*	Plasmid	+	−	+
*ospG^b^*	Plasmid	+	−	+
*virF*	Plasmid	+	+	+
*virG*	plasmid	+	+	+

^*a*^ A + indicates virulence gene was present with a TBLASTN BSR >0.80, while a ± indicates a virulence gene present >0.60, and − is equivalent to any genes <0.60.

^*b*^ Verified by National Center for Biotechnology Information nucleotide megablast and PCR.

^*c*^ Gene *iutA* from M301T (2a) reference strain (AE005674.2:3825260–3826597); CCH060+ for EIEC and ExPEC *iutA* gene (Data Set 2).

^*d*^ PAI, pathogenicity island.

### Genomic variation among with *S. flexneri* archetype virulence plasmids

Whole-genome sequencing also revealed variation in the size of the virulence plasmids between the three archetype strains, with *Sf3*a pJ17B_264 being the largest (~265 kb) and *Sf*6 pCCH060_195 being the smallest (~195 kb) (Table 1). The variation in plasmid size, combined with the observed genomic differences among the plasmid virulence genes (Table 2), led us to evaluate the total genomic content among the virulence plasmids from the archetype strains. Acquisition and maintenance of the pINV are critical for *Shigella* pathogenesis (55), yet comparative analysis of pINV from different *S. flexneri* serotypes has been limited to date (49, 56).

**FIG 3:**
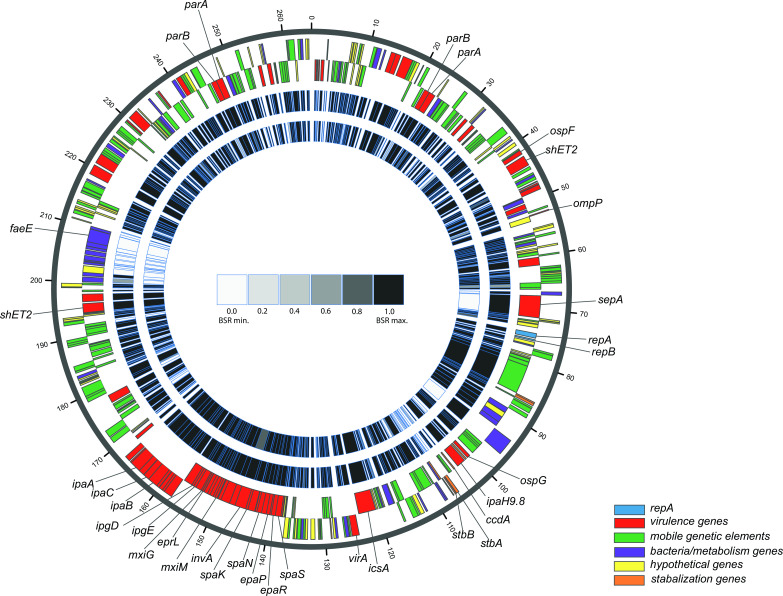
*In silico* analysis of the *S. flexneri* virulence plasmids. Comparison of *S. flexneri* virulence plasmid in *S. flexneri* archetype strains. Protein-coding genes were predicted and annotated in each of the *S. flexneri* archetype strain plasmid using in-house IGS software (59, 60). Gene content of archetype strains pJ17B_264 virulence plasmid to p2457T_221 and pCCH060_195 was analyzed using LS-BSR (52). The genes that were detected with significant similarity are indicated by black, while those identified with divergent sequences are indicated by white (see LS-BSR scale). The outermost rings indicate pJ17B_264, the largest archetype virulence plasmid (Table 1). Each protein-coding ORF is represented by a color-coded rectangle whose size correlates to bp length: virulence (red), mobile elements (green), bacteria/metabolism (purple), hypothetical (yellow), stabilization (orange), and *repA* (blue), of which the outer ring (first ring) contains minus genes and the inner ring (second ring) plus genes. The two inner grayscale rings indicate the BSR value for the corresponding gene in pJ17B_264 (p2457T_221, third ring; pCCH060_195, fourth ring). The full data set can be found in dData Set 2.

The archetype pINV was subjected to LS-BSR analysis utilizing each of the three plasmids as the reference (Fig. 3, Data Set 2). Approximately 80% of the virulence plasmid content is conserved between the three archetype strains (Fig. 3), consistent with recent analysis (57). This level of conservation is irrespective of the archetype reference plasmid used, indicating that there is a conserved plasmid core (Table S2). Our analysis also revealed unique features in the pINV within each of the archetype strains. *Sf*2a strain 2457T contains several unique plasmid stability genes (*stbA*), whereas *Sf*3a strain J17B contains several fimbria subunits. While *Sf*6 pCCH060_195 is the smallest of the virulence plasmids (Table 1), it encodes the greatest number of unique genes, including several transposases, *tonB*-family genes, which could indicate plasmid-chromosome cross-talk for the transport of ferric chelates (58), and hypothetical proteins with unknown function (dData Set 2).

### Exposure to bile salts causes differential transcriptomic profiles in *S. flexneri* strains 2457T, J17B, and CCH060

Exposure to bile within the small intestine has been shown to increase virulence gene expression in 2457T and is thought to prime *Shigella* for colonic infection (25, 61); however, the effect of bile salts on *S. flexneri* serotypes 3a and 6 has not been described. Based on the genomic variation between the three archetype strains, we wanted to determine the bile salt-induced transcriptional profiles among the *S. flexneri* archetype strains.

**FIG 4:**
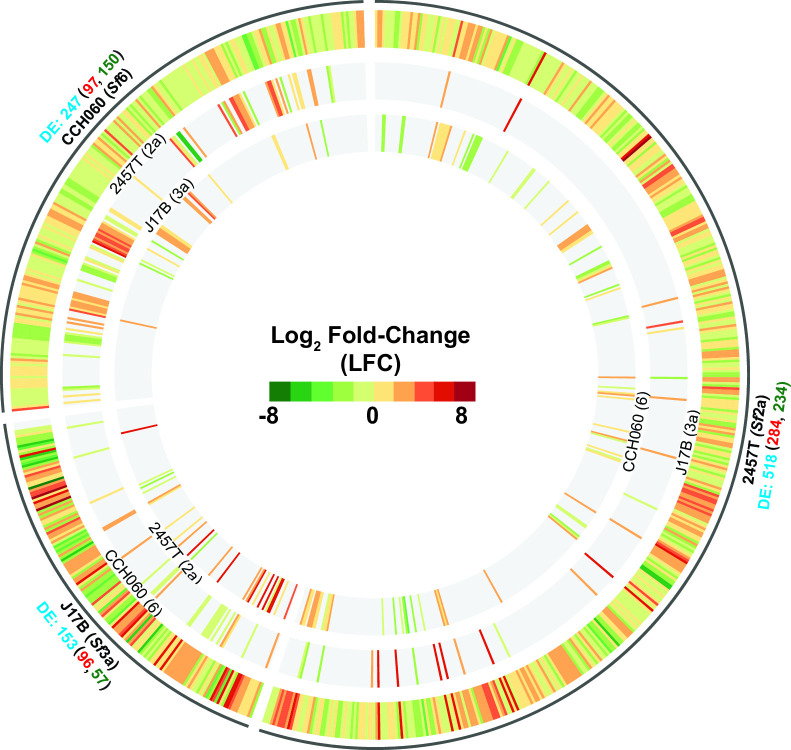
Comparative transcriptomics of the *S. flexneri* archetype strains. Comparison of differentially expressed genes in response to bile salts for each of the *S. flexneri* archetype strains. The serotype of each strain is in parentheses adjacent to the strain name. The total number of differentially expressed (DE) genes of each strain in response to bile salts is indicated in blue below each strain label; the total number of genes with increased expression and decreased expression is indicated in red and green, respectively. The DE genes for each archetype strain are displayed in the outer ring, spanning the black semicircle for each archetype strain. The two inner rings beneath each archetype strain compare the log fold change for each DE expressed strain-specific gene (indicated in blue below each strain label) to the other archetype strains.

Transcriptomic analysis identified differences between *S. flexneri* strains 2457T, J17B, and CCH060 when grown with or without bile salts (Fig. 4). Strain 2457T has the greatest transcriptional response to bile salts with 518 genes differentially expressed (Fig. 4) compared to archetype strain J17B (153 genes) and CCH060 (247 genes). Interestingly, there are no consistent shared differential expression patterns between the archetype strains (Fig. 4). Bile salts appear to have the greatest effect on transcription of genes within the core, or shared, genome (640 genes, Data Set 3). Of the 640 differentially expressed core genes, only two genes have the same differential expression pattern in all three archetype strains, *tehA* encoding tellurite resistance and *ric* encoding an iron-sulfur repair di-iron protein (Table 3, Data Set 3).

**TABLE 3:** Differences in gene expression among the core genome in *S. flexneri* archetype strains^*a*^

Gene categories	Gene expression pattern	
	Upregulated	Downregulated
Strain-specific		
2457T	177	160
CCH060	30	74
J17B	52	15
Shared by two strains		
2457T and J17B	8	1
2457T and CCH060	40	25
J17B and CCH060	4	1
Shared by three strains		
2457T, CCH060, J17B	2	0

^*a*^ All genes compared within this analysis are from the core genome as indicated by LS-BSR (52, 62). Gene categories are based on variation or similarity within DE patterns of core genome genes with a minimum of 2 log fold change (LFC).

Analysis of the bile salt affected core genes for CCH060 revealed that 52.6% (40 of 76 upregulated genes) have shared differential expression with 2457T; 5.2% (4 of 76 genes) shared expression with J17B; and 39.5% (30 of 76 genes) are uniquely affected in CCH060 alone (Table 3). The same relationship is not seen within the 123 downregulated genes in CCH060’s genome; 20.3% (25 of 123) shared with 2457T; 0.8% (1 of 123) shared with J17B; and 60.2% (74 of 123) were uniquely affected in CCH060 alone (Table 3). Several of the differentially expressed genes play a role in metabolism; for example, nickel transport and nitrate reductase genes were upregulated in 2457T but were downregulated in CCH060 (Data Set 3). Unique metabolic factors that were differentially expressed in CCH060 alone include the upregulation of histidine biosynthesis and the downregulation of the nuo-operon (NADH:ubiquinone oxidoreductase, Data Set 3).

### *S. flexneri* strains 2457T and J17B are more invasive and induce greater IL-8 expression than CCH060

**FIG 5:**
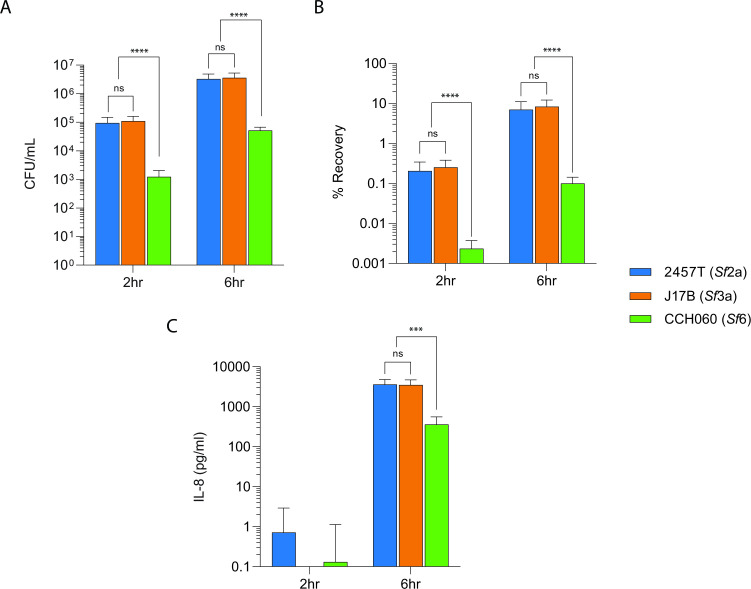
Archetype invasion, intracellular replication, and IL-8 induction vary between *S. flexneri* archetype strains. (**A and B**) *S. flexneri* archetypes were used to infect HT-29 monolayers (MOI of 1:100) for 90 min. Cells were lysed to enumerate intracellular bacteria at 2 and 6 hours pi. Results are displayed as CFU/mL (A) and normalized by inoculum and displayed as percent recovery (B). (**C**) Supernatants were collected at the indicated time points during HT-29 infection (**A and B**). Concentrations of IL-8 were quantified by enzyme-linked immunosorbent assay. Data are normalized by subtraction of uninfected controls and are presented as pooled from three independent experiments (*n* = 3). The asterisks above indicate statistically significant differences determined using a two-way analysis of variance and Tukey post-test. ***P < 0.001, ****P < 0.0001. ns, not significant.

The genomic diversity of the archetype strains prompted us to evaluate the variation in characteristic virulence phenotypes. A gentamicin protection assay was used to assess the ability to invade and replicate intracellularly in HT-29 epithelial cells at 2 and 6 hours post-infection (pi) (30). At 2 hours pi, the intracellular recovery of CCH060 was significantly less than the other two archetype strains (Fig. 5A and B). CCH060 recovery was 0.002%, a ~100-fold decrease (*P* < 0.0001) compared to 2457T (0.20%) and J17B (0.25%) (Fig. 5B). The lower CCH060 recovery at 6 hours pi is not the result of reduced intracellular replication, as all three archetype strains underwent a similar number of doublings between 2 and 6 hours (5.4 CCH060, 5.7 2457T, and 5.3 J17B). Instead, the reduced invasion appears to be a result of a CCH060 strain-specific feature as the intracellular recovery can be rescued by increasing the multiplicity of infection (MOI) fourfold (Fig. S1)

In addition to invasion, we measured IL-8 secretion from HT-29 cells, a pro-inflammatory cytokine which is a hallmark of *Shigella* infection (40). CCH060 induced a statistically reduced amount of IL-8 at 6 hours pi, which is approximately 10-fold less than strains 2457T and J17B (Fig. 5C). There was no difference at 2 hours pi as levels of cytokine release were too low to measure.

### *S. flexneri* strain CCH060’s decreased invasion phenotype is not affected by bile salts nor adherence to HT-29 cells

**FIG 6:**
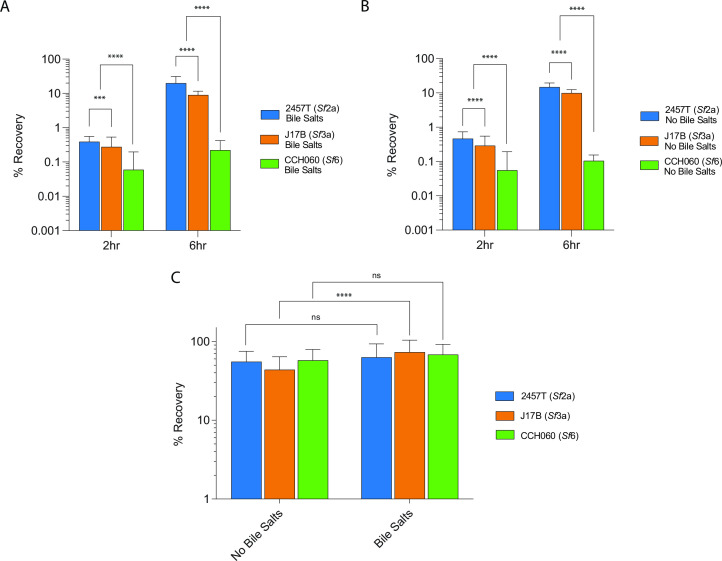
Effect of bile salts on adherence and invasion in archetype strains. (**A and B**) *S. flexneri* archetype strains were grown with (**B**) or without (**A**) bile salts to log phase (OD_600_, ~0.7) then used to infect HT-29 monolayers (MOI of 1:10) for 90 min. Cells were lysed to enumerate intracellular bacteria at 2 and 6 hours pi. The data presented are pooled from three independent experiments (*n* = 3). (**C**) *S. flexneri* archetype strains grown with or without bile salts were used to infect HT-29 monolayers (MOI of 1:10) for 90 min. Cells were washed vigorously and lysed to enumerate intracellular and adherent bacteria at 2 hours. Adherence was calculated by subtracting intracellular bacteria from the corresponding treatment condition (**A and B**) from the total recovered bacteria without gentamicin treatment. The data presented are pooled from five independent experiments (*n* = 5). The asterisks above indicate statistically significant differences determined using a two-way analysis of variance and Tukey post-test. ***P < 0.0001, ****P < 0.0001. ns, not significant.

Based on the differential bile-induced transcriptional profiles between the archetype strains, we examined the effects of bile salts on invasion (Fig. 5). Previous studies with strain 2457T report conflicting results regarding the invasion and adherence phenotype in response to bile salts (61, 63). In our studies, the archetype strains were grown in tryptic soy broth (TSB) alone or TSB containing 0.1% deoxycholate (DOC) for ~2 hours, prior to infection of HT-29 cells. The addition of bile salts had no effect on the reduced invasion phenotype of CCH060 (Fig. 6A and B); CCH060’s intracellular recovery remained ~10-fold less compared to 2457T and J17B at 2 hours pi. The CCH060 recovery was 0.02% of the initial inoculum, compared to 0.29% for 2457T and 0.21% for J17B. There was no statistical difference in invasion between bacteria grown in TSB alone vs TSB with 0.1% deoxycholate.

*Shigella* invasion is dependent on host cell contact via the T3SS, which plays a role in both adherence and secretion of effector proteins (36). While adherence alone does not dictate invasion levels (64), as these are often considered to be independent events (63), with greater adherence, the T3SS effectors may be able to be delivered more efficiently, thus enhancing cytoskeleton remodeling. We hypothesized that CCH060 may have a reduction in adherence compared to the other archetype strains that could affect its ability to be internalized into HT-29 cells. The adherence levels of each archetype strain, grown with and without bile salts, were assessed at 2 hours pi with HT-29 cells. There was no statistically significant difference between the archetype strains in either growth condition (Fig. 6C). Examining the effect of bile salts on adherence for an individual archetype strain between conditions, we observed that J17B was the only strain to adhere at greater levels with exposure to bile salts. This is in contrast to previous studies that have shown that exposure to bile salts results in increased adherence in *Sf*2a and *Sf*5 strains (61, 63). Methodological differences, such as greater MOI and growth conditions of the culture, may contribute to the observed differences between studies. Overall, these results suggest that CCH060’s decreased intracellular recovery in TSB alone or TSB with bile salts is not due to a difference in adherence between the archetype strains.

### CCH060’s decreased intracellular recovery may be a result in variation in T3SS effector proteins

**FIG 7:**
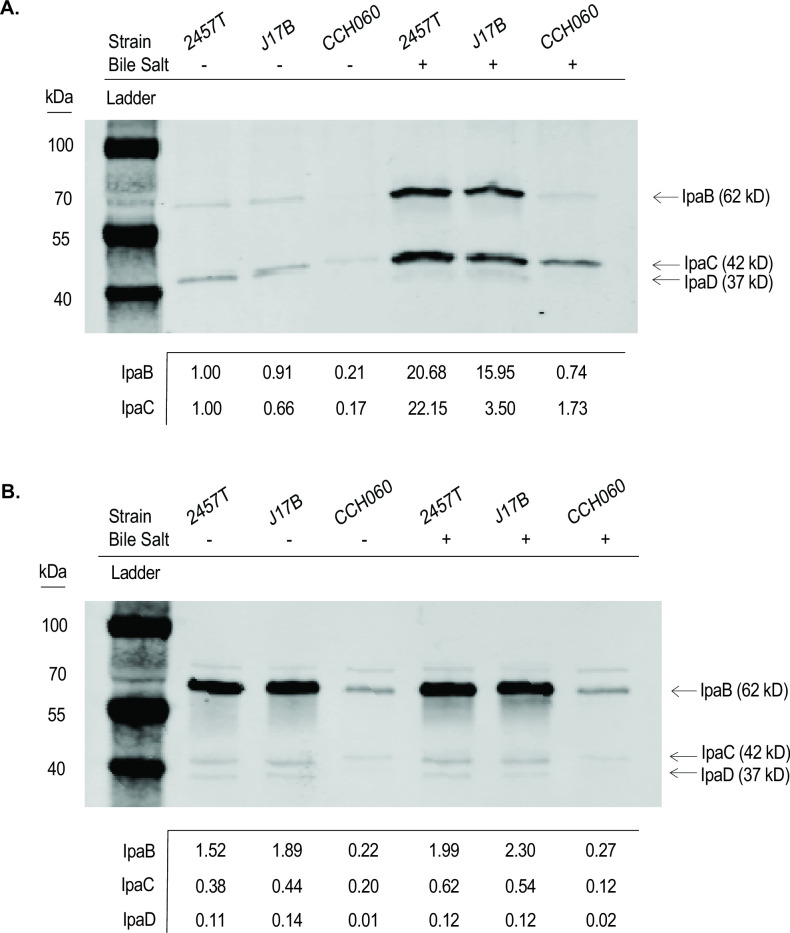
CCH060 has reduced whole cell and secreted levels of Ipa protein expression compared to 2457T and J17B. (**A**) Supernatant and (**B**) whole cell lysates collected during TSB (−) and TSB with bile salt (+) growth conditions were probed for IpaB, IpaC, and IpaD by Western blot. Each archetype strain and growth condition were collected at mid-log phase (OD_600_, ~0.7, 2 × 10^8^ CFU/mL). Whole cell lysates were normalized using DnaK (Invitrogen) as a loading control. Secreted protein fractions were assessed by assuming 2457T TSB− was 1 and comparing all secreted levels protein levels to it. Densitometry (ImageJ) was utilized to compare loaded protein levels (66). Total protein levels for supernatants and whole cell lysate controls were used to confirm if the effect of bile salts on *S. flexneri* archetype isolates was consistent with previous literature (61) (Fig. S2).

The T3SS is critical for *Shigella* internalization into epithelial cells via secretion of effector proteins, such as the Ipa proteins. To assess T3SS effector secretion by archetype strains, we used bile salts to stimulate secretion (61, 65). Secreted as well as whole cell protein levels of IpaB, IpaC, and IpaD were compared. Bile salt exposure resulted in an increase in total protein secretion (Fig. S2A), as well as increased secretion of IpaB, IpaC, and IpaD (Fig. 7A) for all three archetype strains compared to those not exposed to bile salts. This result is consistent with previous results for *S. flexneri* 2a protein secretion induction assays (61). Of the three archetype strains, CCH060 demonstrated the lowest secretion levels of Ipa proteins (Fig. 7A). IpaD secretion, while visually decreased for CCH060, did not appear significant as levels were too low to accurately quantify using densitometry. Importantly, there was no increase in Ipa protein expression in whole cell lysates as a result of deoxycholate exposure (Fig. 7B; Fig. S2B). Instead, the observed reduction in secreted Ipa protein by CCH060 is a result of reduced whole cell levels of Ipa protein.

The reduced protein levels of Ipa protein for CCH060 may be a result of variation in transcription and/or protein-protein interactions. Using qRT-PCR to compare strain-to-strain transcriptional variation, we identified a significant reduction in *ipaC* and *ipaD* transcript levels, but not *ipaB* transcripts for CCH060 (Fig. S3). While *ipa*B transcripts for CCH060 do not mirror reduced protein levels, this analysis does not rule out *Sf*6-specific transcription regulators or protein-stability variation.

Another explanation for reduced intracellular protein recovery could be directly related to modifications in protein structure and function. CCH060 had the greatest nucleotide polymorphisms of the three archetype strains compared to *ipa* gene references from *S. flexneri* serotype 2a strain 301T (GCA_000006925.2); *ipaB* from CCH060 was 98.5% identical to the reference (2457T 100%, J17B 99.7%); *ipaC* was 97.5% identical (2457T 99.9%, J17B 99.6%); and *ipaD* was 97.4% identical (2457T 100%, J17B 99.5%). The nucleotide changes in CCH060 result in amino acid substitutions that could alter protein function. We observed several conserved amino acid substitutions within the coiled-coil regions among these three proteins for CCH060 as compared to references from *S. flexneri* serotype 2a strain 301T (Fig. S4): IpaB variation in the C-terminal residues 110–170 (Fig. S4A), IpaC variation in the C-terminal domain III residues 261–363 (Fig. S4B), and IpaD variation in the globular domain residues 179–275 (Fig. S4C). Previous studies have demonstrated that mutations in these regions (deletions or single amino acid substitutions) result in the modification of translocon formation, cytotoxicity, and intracellular recovery (67–72).

## DISCUSSION

The genomic and phenotypic diversity of *Shigella* isolates is a significant hurdle in development of a broadly protective vaccine (10). GEMS and other multicenter studies have confirmed the importance of *S. sonnei* and *S. flexneri* 2a, *S. flexneri* 3a, and *S. flexneri* 6 as predominant species and serotypes causing disease and have suggested a quadrivalent vaccine strategy that would provide 89.4% coverage based on homologous and cross-reacting O-antigens (2). Recent genomic analyses have revealed significant genomic heterogeneity within and between the *S. flexneri* serotypes (17), which may provide further direction for vaccine development strategies (12). Moreover, given the genomic diversity between the *S. flexneri* serotypes, the assumptions regarding the *Shigella* pathogenesis phenotype may not be universal to all serotypes. In this study, we used genomic, transcriptomic, and phenotypic analyses of representative archetype strains of the three most prevalent *S. flexneri* serotypes implicated in disease in LMICs (7) to understand how they differ from one another.

We completed the genomes of the archetype strains 2457T (*Sf*2a), J17B (*Sf*3a), and CCH060 (*Sf6*) for use as references in this and future studies. Each of the three strains contains a single chromosome, as well as a version of the ~200-kb pINV, *Shigella* virulence plasmid. Our comparative genomic analysis of the archetype strains highlighted that, while much of the genome is conserved, there are strain-specific genomic features as well as variations in established *Shigella* virulence genes. Of the three archetype strains, *Sf*6 strain CCH060 contained the greatest amount of unique genomic content and was genomically distant from 2457T and J17B, consistent with the limited previous *Sf*6 studies (17). The significant genomic differences of CCH060 suggested that there may unique genomic features that may confer strain-specific phenotypic patterns. One set of genes we identified that may confer a phenotypic advantage encodes a T2SS which, to date, has not been identified in *S. flexneri* nor has a role in virulence assigned within *Shigella*. In *E. coli*, the T2SS plays a role in virulence and survival (73), and two forms of the T2SS have been identified within divergent *E. coli*: T2SS_α_ and T2SS_β_ (74). An assessment of homology revealed the T2SS intact genes, unique to CCH060, are 96.7% genetically identical to *E. coli* T2SS_β_ (ETEC-T2SSB) in reference strain H10407 (Data Set 2). Whether this secretion system is functional or plays a role in *S. flexneri* serotype 6 virulence is currently under study.

While unique genomic features may confer phenotypic variations, so too can gene loss as has been demonstrated in *Shigella* (75). Our *in silico* analysis revealed that of the three archetype strains, CCH060 is lacking several chromosome- and plasmid-located established virulence genes. The virulence gene loss supports our hypothesis that CCH060 may have an altered phenotype compared to other *S. flexneri* serotypes; for example, the absence of *sepA* could affect basolateral invasion through decreased loosening of tight junctions (76), while the absence of *ospG* (77) or *shiA* (78) has been shown to cause a hyperinflammatory response in the host resulting in increased bactericidal activity. Given the extent of unique genomic content, it is also likely that there are additional novel *S. flexneri* serotype 6-specific virulence factors that have yet to be identified and characterized.

*Shigella* spp. are able to survive the bactericidal effect of bile salts in the gastrointestinal tract and have been documented to use bile salts as an environmental stimulus resulting in modulation of transcriptional and virulence activity (25, 61, 63, 65). To date, the effect of bile salt on the transcriptomic profile for *S. flexneri* has not been evaluated outside of *S. flexneri* 2a strain 2457T (25). Our results demonstrate that bile salts affect each archetype stain differently, yielding a strain-specific response, causing the greatest differential expression within 2457T. Comparison of the transcriptome to whole-genome content revealed that the greatest differential expression was that within the core genome, however, only two genes exhibited a shared expression pattern between all three of the archetype strains under the same bile stimuli. This was surprising, as we anticipated there would be strain-specific genomic features that were differentially affected but that the core genome response would be conserved. These strain-specific transcriptional responses may result in significant differences outside of the limited virulence-associated phenotypes explored in this study. At minimum, our results suggest that in order to account for these transcriptomic differences, bile salts should be considered in the laboratory to better represent *in vivo*-like environments when comparing infection models of *S. flexneri*.

Evaluation of critical *Shigella* virulence phenotypes between archetype strains revealed that CCH060 invaded HT-29 cells significantly less than 2457T and J17B and induced less IL-8 production. The decreased intracellular load was neither a result of deficiencies of CCH060 intracellular replication, as all three strains underwent a similar number of doublings, nor a result of decreased adhesion. The reduced invasion phenotype was consistent despite variation in growth conditions (agar plate grown, logarithmic growth in broth) or the addition of stimuli, such as bile salts. The differences in growth conditions affected bacterial recovery for CCH060; logarithmic phase bacteria invaded ~100-fold more than ager plate-grown bacteria. Previous studies have identified unique metabolic processes for *Sf*6, not found in serotypes 1–5 (22) or through biochemical analyses (79). Our studies revealed numerous metabolic factors that are only stimulated in CCH060 with the addition of bile salts, which may account for this variation (Data Set 2).

The reduced invasion of CCH060 compared to the other strains might suggest reduced virulence. However, *Sf*6 is responsible for significant disease burden, and the clinical disease is indistinguishable between the serotypes. We showed that by increasing the inoculum fourfold, the reduction in invasion efficiency could be rescued. The data suggest that while unique genomic features may be playing a role in pathogenesis, the differences in recovery may be due to structural or functional differences in the T3SS.

Western blots revealed that both secreted and whole cell levels of IpaB, C, and D were greatly reduced in *S. flexneri* serotype 6 strain CCH060 compared to archetype strains 2457T and J17B. These data suggest that CCH060’s reduced intracellular recovery could be due to a reduction in total effector protein levels. Additionally, evaluation of *ipa* transcripts identified a significant reduction in *ipaC* and *ipaD* transcripts but not *ipaB* for CCH060 as compared to the other archetype strains. IpaD plays a critical role in the recruitment of IpaB/IpaC to the T3SS tip to form the translocon (80) and acts as a scaffold, attaching the MxiH portion of the needle to the pore forming IpaB and IpaC proteins (81). Examination of the primary amino acid sequence of IpaD identified several amino acid substitutions within the C-terminal globular domain, which could affect IpaB-IpaD binding. It is possible that reduction in IpaD and protein sequence variations could affect cytosolic IpaB levels as well as reduce the number T3SS translocon needles on the surface, thus reducing the number of host-membrane association opportunities. Further analysis of primary amino acid sequences for IpaB and IpaC identified differences in IpaB and IpaC C-terminal coil-coil domains for CCH060. These proteins sit at the periphery of the bacteria membrane to directly interact with the host cell membrane (36). Variation in host-membrane association through either recognition or interaction with target cell cytoskeleton could also account for reduced intracellular recovery. Collectively, these data suggest that reduced transcription of *ipa*C and *ipa*D resulting in reduced protein levels of IpaB, IpaC, and IpaD together with amino acid substitutions affect the efficient assembly and function of the T3SS and ultimately reduced invasion. Further studies utilizing mutational analysis and protein functional studies will resolve these functional questions in the future.

Overall, our analysis identified genomic, transcriptomic, and phenotypic differences among commonly used archetype strains for the most prevalent *S. flexneri* serotypes. These are data which have been lacking in the literature. The most significant differences were identified in the *Sf*6 archetype strain CCH060. These findings will ultimately influence the design and efficacy of target interventions and vaccines against *S. flexneri* targeted interventions and support future studies to identify conservation of features among a broad collection of *Sf*6 isolates.

## MATERIALS AND METHODS

### Bacterial strains and growth conditions

*S. flexneri* strains were grown in TSB (logarithmic growth) or on tryptic soy media (TSA) containing agar (BD Difco). Congo red (Sigma-Aldrich, St. Louis, MO) was added to TSA media at a final concentration of 0.01% (wt/vol) and supplemented with 0.005% guanine to make CR-TSA plates; agar plate grown (45). Bile salt-induced strains were grown in tryptic soy broth (Sigma-Aldrich) containing 0.1% (wt/vol) DOC (Sigma-Aldrich), logarithmic grown (65).

### Growth curves

Red only colonies for each strain were inoculated in 100-mL TSB or 100-mL TSB containing 0.1% deoxycholate and were grown for 2 hours. The optical density at 600 nm (OD_600_) and enumeration CFU per milliliter were taken every 30 min. Serial dilutions were plated in quadruplicate on CR-TSA plates and incubated overnight at 37°C to determine CFU per milliliter (Fig. S5).

### Genome sequencing and assembly

The representative *S. flexneri* strains were grown in lysogeny broth (LB) overnight, and their DNA was purified using a modified alkaline lysis and phenol-chloroform extraction method (82). The purified DNA was used to construct libraries and was sequenced on the Illumina HiSeq 4000 and the Pacific Biosciences RS II with P6C4 chemistry and were assembled as previously described (82).

### Phylogenomic analysis

The genomes of *S. flexneri* 2457T, J17B, and CCH060 were compared with 79 previously sequenced *S. flexneri* genomes (Table S1) and 37 diverse *E. coli* and *Shigella* spp. genomes (83). SNPs were determined against reference genome *E. coli* strain IAI39 (GenBank accession no. NC_011750.1) using NASP v.1.2.0 (84) with default parameters. There were 197,524 conserved SNPs relative to the reference genome. SNPs were used to infer a maximum-likelihood phylogeny using IQ-TREE 2 (85), with the generalized time-reversible (GTR) site substitution model with discrete GAMMA (Γ) distributed rate variation and the Lewis ascertainment bias correction (ASC_GTRGAMMA) and 100 bootstrap pseudoreplicates. The phylogeny was midpoint-rooted and decorated using FigTree v.1.4.2 (http://tree.bio.ed.ac.uk/).

### Gene-based comparisons

We investigated differences in the total gene content among the genomes of *S. flexneri* 2457T, J17B, and CCH060 using BLASTN LS-BSR analysis as previously described (52, 86). The protein-coding genes of each genome were assigned to gene clusters with ≥90% nucleotide identity and ≥90% alignment length using CD-HIT v.4.6.7 (87) (Data Set 2). Gene clusters identified with a BSR value of ≥0.8 were considered present with significant similarity, while gene clusters with a BSR value <0.4 were considered absent. The Venn diagram indicates the gene clusters present in all three genomes, only two genomes, or present in only one of the genomes.

### *In silico* detection of plasmids and virulence genes

Plasmid incompatibility types were identified in each *Shigella* genome using the PlasmidFinder v.1.3 database (88). Plasmids in each of the complete genomes were annotated using an in-house annotation pipeline (59, 60). To analyze genomic variation between the plasmids, LS-BSR analysis was used as previously described utilizing each respective plasmid as a reference (52, 86). The circular plot was generated as previously described (83, 89) using Circos v.0.69-6 (90). *Shigella* virulence genes (Data Set 2) were detected in *S. flexneri* 2457T, J17B, and CCH060 using TBLASTN LS-BSR as previously described (52, 86).

### RNA sequencing and analysis

*S. flexneri* strains 2457T, J17B, and CCH060 were grown in TSB alone or TSB supplemented with 0.1% DOC by inoculating five red colonies from overnight CR-TSA plates. Biological duplicates were incubated at 37°C shaking at 250 rotations per minute (rpm) for ~2 hours until the OD_600_ reached mid-log phase (OD ~0.7). Cultures were centrifuged at 4,500 rpm at room temperature for 5 min. The supernatant was removed and the cell pellets were suspended in 500 µL TriPure isolation reagent (Sigma-Aldrich). The TriPure manufacturer protocol was used to isolate total RNA from the bacterial cultures. TURBO DNase (Thermo Fisher Scientific, Waltham, MA, USA) was used to treat the samples and remove genomic DNA. The RNA was sequenced and analyzed as previously described (62, 83, 89, 91). The paired RNA-seq reads were aligned to each respective genome using Bowtie (92). The number of reads that aligned to each protein-coding gene were compared between the bile and no bile control samples for each strain using DESeq (93). The predicted protein-coding genes of each genome were identified using an in-house annotation pipeline (59, 60). The protein-coding genes of the *S. flexneri* 2457T, CCH060, and J17B genomes were compared using CD-HIT v.4.6.8 (87) to generate gene clusters with ≥90% nucleotide identity and ≥90% alignment length. The gene clusters were used to identify genes present in all three genomes, only two genomes or present in only one of the genomes (Data Set 3). These data were used to assess the prevalence of the differentially expressed genes of each archetype strains. The circular plot was generated as previously described (83, 89) using Circos v.0.69-6 (90).

### HT-29 cell cultivation and gentamicin protection assay

Human HT-29 (ATCC HTB-38) monolayers were cultured in DMEM (Corning) supplemented with 10% Fetalplex (Gemini) and 2% HEPES (Quality Biological) in 150 cm^2^ flasks (Corning). The cells were incubated in 5% CO_2_ at 37°C and passaged as required.

#### Non-bile salt gentamicin protection assay: agar plate-grown

HT-29 cells were seeded at a density of 6 × 10^5^ cells per well in a 24-well plate and incubated overnight at 37°C with 5% CO_2_(Fig. 5). Red colonies were picked from overnight plates and resuspended in Dulbecco’s phosphate-buffered saline (DPBS) to a concentration of 1 × 10^8^ CFU/mL. One milliliter of inoculum at an MOI of 1:100 was added to the HT29 monolayers in triplicate for each strain. Infected monolayers were centrifuged for 5 min at 3,000 × *g* to enhance bacteria-cell contact then were incubated at 37°C with 5% CO_2_ for 90 min to allow for bacterial invasion of HT29 monolayers. Following 90 min invasion, the infected monolayers were washed with PBS and incubated with DMEM containing 50 µg/mL gentamicin 30 min at 37°C with 5% CO_2_ to remove extracellular bacteria. This is considered the 2-hour post-infection time point. To assess intracellular replication, the monolayers were incubated in gentamicin-containing medium for an additional 4 hours (considered 6 hours post-infection, respectively). The media were collected for cytokine analysis. To enumerate intracellular bacteria, the monolayers were washed with DPBS and lysed using 1% Triton X-100. Serial dilutions were plated in quadruplicate on CR-TSA plates and incubated overnight at 37°C. Percent recovery was calculated using the following formula: recovered bacterial titer/infecting bacterial titer × 100% (25, 45, 94). The number of doublings was calculated between 6 hours pi (T6) and 2 hours pi (T2) using the following formula: log_10_T6 CFU – log_10_T2 CFU) × 3.32 (94).

#### TSB/bile salt gentamicin protection assay: logarithmic growth

Red colonies were picked from overnight plates and resuspended in TSB or TSB supplemented with 0.1% DOC, to a concentration of ~6 × 10^7^ CFU/mL (OD_600_ of 0.1) (Fig. 6). Bacteria were incubated at 37°C for ~2 hours to mid-log phase (OD_600_ of ~0.7; 2 × 10^8^ CFU/mL). Bacteria were centrifuged at 4,500 × *g* for 10 min to pellet the bacteria. Bacterial pellets were washed in 1× PBS, centrifuged at 4,500× *g* for 10 min again to remove residual bile salts that could compromise the HT-29 monolayers. Due to the variation in growth conditions, 2457T lysed HT-29 at an MOI of 1:100; thus, bacteria were resuspended in DMEM at an MOI of 1:10 (1 × 10^8^ CFU/mL), and the gentamicin protection assay protocol was followed as described above.

### Adherence assay

To analyse adherence, HT-29 cells were seeded at a density of 6 × 10^5^ cells per well in a 24-well plate in duplicate and incubated overnight at 37°C with 5% CO_2_. Bacteria were grown to mid-log in TSB or TSB supplemented with 0.1% DOC as described above. Duplicate HT-29 plates were infected in triplicate for each archetype strain, and gentamicin protection assay was performed as described above with the exception that one plate was treated with no gentamicin after 90-min incubation. To determine the number of bacteria that invaded vs adhered to the monolayers, serial dilutions were performed to determine the CFU per milliliter recovered for gentamicin treated and untreated HT-29 plates. The number of adhered bacteria was calculated by subtracting the intracellular bacterial load (gentamicin treated HT-29 plate) from the total bacteria recovered (DMEM only HT-29 plate).

### Cytokine analysis

The supernatant media from the gentamicin protection assays were collected for cytokine analysis using a DuoSet enzyme-linked immunosorbent assay (ELISA) kit for human IL-8 (R&D Systems). The ELISA was performed according to the manufacturer’s protocol. The amount of IL-8 was reported in picograms contained in the total volume (1 mL) of the culture supernatant present at the indicated times post-infection (2, 6, 8, and/or 20 hours).

### Western blot

Western blot samples were collected from qRT-PCR growth collections for consistency. Red colonies were picked from overnight plates and resuspended in TSB or TSB supplemented with 0.1% DOC, to a concentration of ~6 × 10^7^ CFU/mL (OD_600_ of 0.1). Bacteria were incubated at 37°C for ~2 hours to mid-log phase (OD_600_ of ~0.7, 2 × 10^8^ CFU/mL). Bacteria were centrifuged at 4,500 × *g* for 10 min to pellet the bacteria and then filtered (0.2-µm pore size) to separate supernatant from bacteria.

#### Supernatant

One milliliter of supernatant was concentrated with 10% trichloroacetic acid on ice for 1 hour. Samples were centrifuged at 14,000 rpm for 15 min at 4°C to pellet extracted protein. The supernatant was removed, washed with 95% ETOH, and resuspended in 2× Lamelli buffer Sample Buffer (BioRad) containing 10% B-mercaptoethanol.

#### Whole cell lysate

One milliliter of bacteria for each corresponding supernatant was centrifuged at 14,000 rpm for 15 min at 4°C to pellet the bacteria. Supernatant was removed and bacteria were resuspended in 1:1 ratio of dH_2_O and 2× Lamelli buffer (BioRad) containing 10% β-mercaptoethanol. Samples were then heated at 95°C for 10 min for protein denaturation.

#### Total protein

Proteins were separated on a 12% Mini-Protean TGX Precase Gel (BioRad) before being incubated overnight in GelCode (Thermo Fisher). Gels were washed with deionized water and imaged using GeneSys.

#### Ipa protein visualization

Proteins were separated on a 12% Mini-Protean TGX Precase Gel (BioRad) before being transferred to polyvinylidene difluoride (PVDF) membrane. The membrane was blocked with 10% (wt/vol) non-fat milk buffer in 1× DPBS and then incubated with anti-IpaB, anti-IpaC, and anti-IpaD antibody (1:10,000) (kindly donated by the Wendy Picking Laboratory) diluted in 10% (wt/vol) non-fat milk buffer overnight at 4°C on an orbital shaker. Following washing, the membrane was incubated with secondary goat anti-rabbit 680 nm (Thermo Fisher), and proteins were visualized using 700-nm wavelength setting on a LI-COR Odyssey Laser Scanner. Whole cell lysates were normalized using DnaK (Invitrogen) as a loading control, and densitometry (ImageJ) (66) was utilized to compare loaded protein levels. There is no other defined protein in *Shigella* that is secreted in a consistent quantity for use as a control. Densitometry (ImageJ) for secreted fractions were determined by assuming 2457T without bile salts was 1 and comparing all secreted levels protein levels to it.

### Endpoint PCR

Diagnostic PCR assays were performed to confirm the *in silico* genomic loss detected in *S. flexneri* serotype 6 strain CCH060. Primers used for *ospG*, *shiA*, *sepA*, *sigA*, *gtrII*, and *pic* can be found in Table S4. Template DNA consisted of a single, red bacteria colony added to the reaction mixture. Reactions were performed with parameters specific for the primer length and composition, as well as the length of the product (Table S4).

### qRT-PCR

Archetype strains were grown from TSA-CR plates in TSB to mid-log phase (OD_600_ of ~0.7) at 37°C with agitation. One milliliter of each of each culture was sampled for total RNA isolation. Bacterial pellets were resuspended in TriPure Isolation Reagent (Roche Life Sciences), extracted with chloroform, and precipitated with isopropanol. Each RNA pellet was resuspended in nuclease-free water (Thermo Fisher Scientific) plus 1 µL of RNase Out (Thermo Fisher Scientific). Contaminating DNA was removed from the total RNA sample using the Turbo DNA-free kit (Thermo Fisher Scientific), and the absence of contaminating DNA was confirmed by endpoint PCR of *rpoA*. The cDNA was generated using the qScript cDNA Synthesis Kit (Quantabio). The qPCR primer sequences (Table S4) were generated for *ipaB*, *ipaC*, and *ipaD*. Each gene was analyzed in triplicate from three biological replicate samples using FastStart Universal SYBR Green Master Mix (Rox) (Roche Life Sciences). The housekeeping gene *rpoA* (91) was used to normalize across samples. The fold change of each transcript was calculated using a ΔΔCt analysis (95): the Ct value recorded for each archetype strain was subtracted from the average Ct value of *rpoA* replicated to give the normalized ΔCt value. Archetype strain 2457T in either TSB or TSB-DOC growth conditions was used as the control sample for archetype vs archetype comparisons, and each archetype strain in TSB growth conditions was used as the control for archetype growth condition comparison. The average control value was subtracted from the ΔCt value of each replicate sample to give the ΔΔCt value for each replicate sample. The fold change in each sample was then calculated as 2^−ΔΔCt^, and the average fold change from triplicate wells was then calculated from these values, which allowed for comparison of relative transcript quantity.

### Nucleotide and amino acid alignment

Each archetype *ipaBCD* nucleotide sequence was isolated from the corresponding fasta file (Table S1) and aligned against *ipa* reference (301T, *S. flexneri* serotype 2a) using MAFFT FFT-NS-I v.7.487 (96). Predicted amino acid structures were predicted using EMBOSS Transeq (97) and aligned using Clustal MUSCLE v.3.8 (98). Functional domains are indicated above the sequence based on previous literature (37, 70, 71, 99–101).

### Statistical analyses

Statistical significance for multiple comparisons was determined using analysis of variance with Tukey post-test to determine statistical differences within specific groups. A *P* value of ≤0.05 was considered significant. Replicates from multiple independent replicate experiments were pooled. The number of replicates pooled from “n” independent experiments is reported in the figure legends. GraphPad Prism software was used for all graphical statistical analyses.

## Data Availability

The complete genome assemblies of *Shigella flexneri* 2457T, J17B, and CCH060 are deposited in GenBank under the accession numbers listed in Table 1.

## SUPPLEMENTAL MATERIAL

Data Set 1 msphere.00408-23-s0001.xlsx Gene annotation for archetype strains.

Data Set 2 msphere.00408-23-s0002.xlsx LS-BSR analyses.

Data Set 3 msphere.00408-23-s0003.xlsx RNA-seq analysis.

Figure S1 msphere.00408-23-s0004.pdf MOI challenge.

Figure S2 msphere.00408-23-s0005.pdf Western blot/total protein.

Figure S3 msphere.00408-23-s0006.pdf qRT-PCR.

Figure S4 msphere.00408-23-s0007.pdf Amino acid alignments.

Figure S5 msphere.00408-23-s0008.pdf Growth curve.

Legends msphere.00408-23-s0009.docx Supplemental legends.

Supplemental Tables msphere.00408-23-s0010.docx Tables S1-S4.

ASM does not own the copyrights to Supplemental Material that may be linked to, or accessed through, an article. The authors have granted ASM a non-exclusive, world-wide license to publish the Supplemental Material files. Please contact the corresponding author directly for reuse.
