# Supplementary figures and images for: Genomic, transcriptomic, and phenotypic differences among archetype Shigella flexneri strains of serotypes 2a, 3a, and 6
Source: mSphere. 2023 Oct 13;8(6):e00408-23. doi: 10.1128/msphere.00408-23 (PMC10732043; doi:10.1128/msphere.00408-23)

A

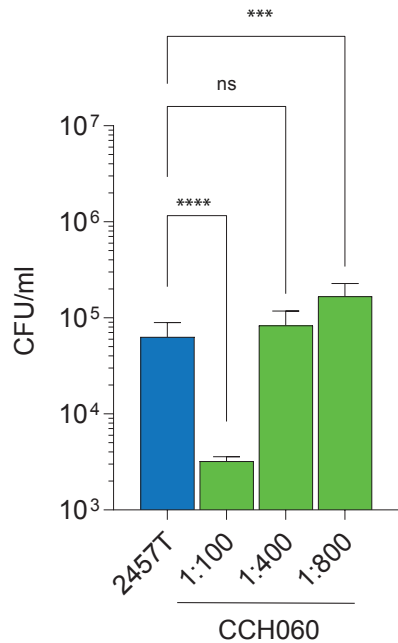

B

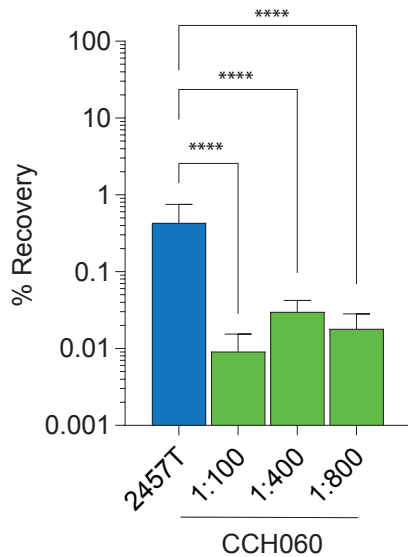

Supplement: Figure S1 — MOI challenge. [file msphere.00408-23-s0004.pdf]

A

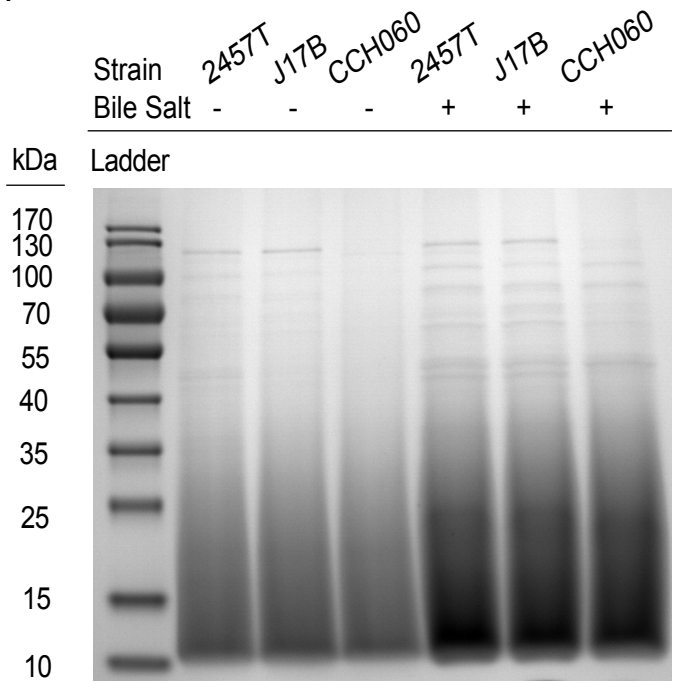

B

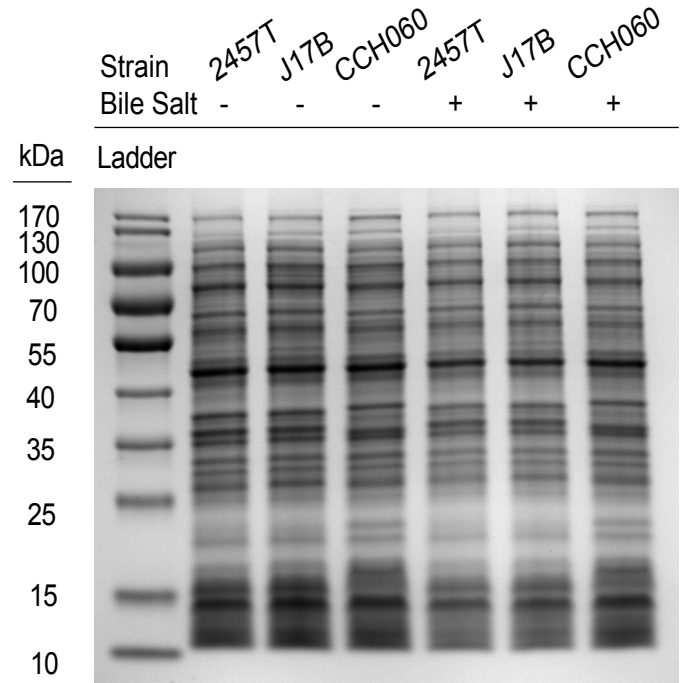

C

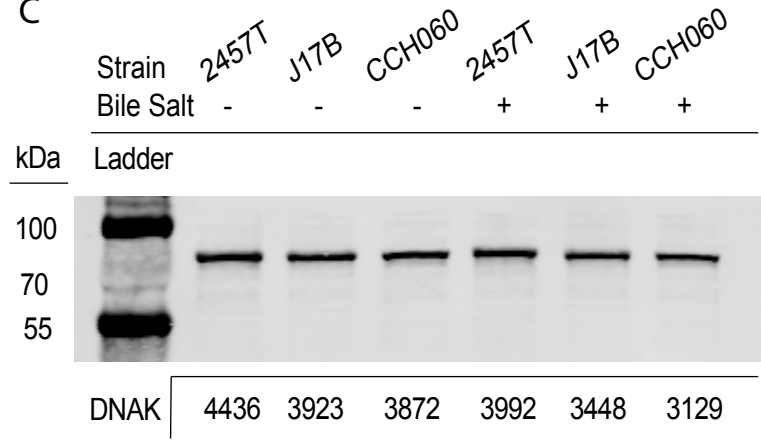

Supplement: Figure S2 — Western blot/total protein. [file msphere.00408-23-s0005.pdf]

A

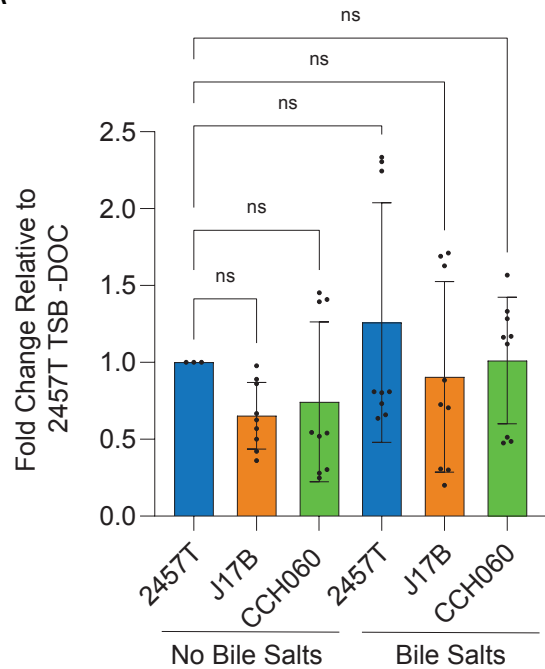

B

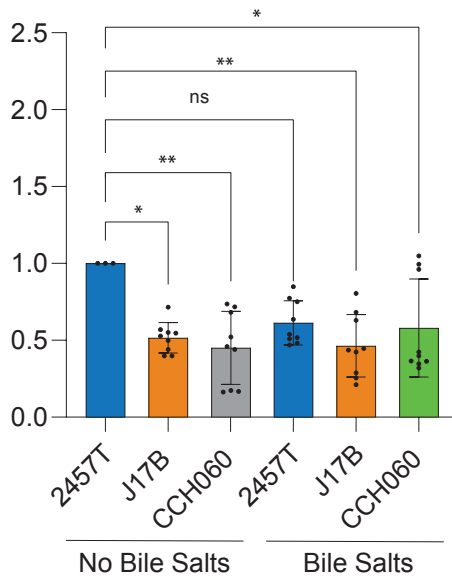

C

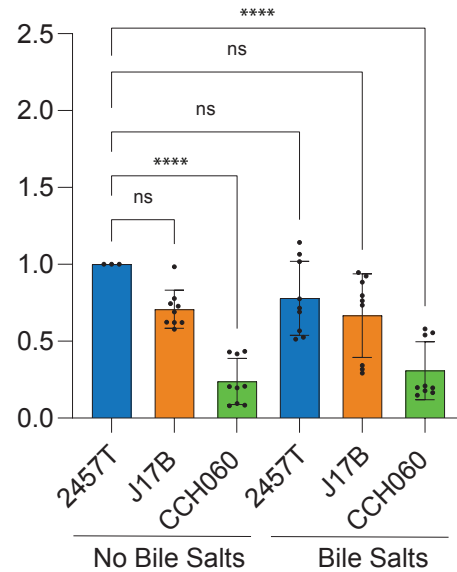

Supplement: Figure S3 — qRT-PCR. [file msphere.00408-23-s0006.pdf]

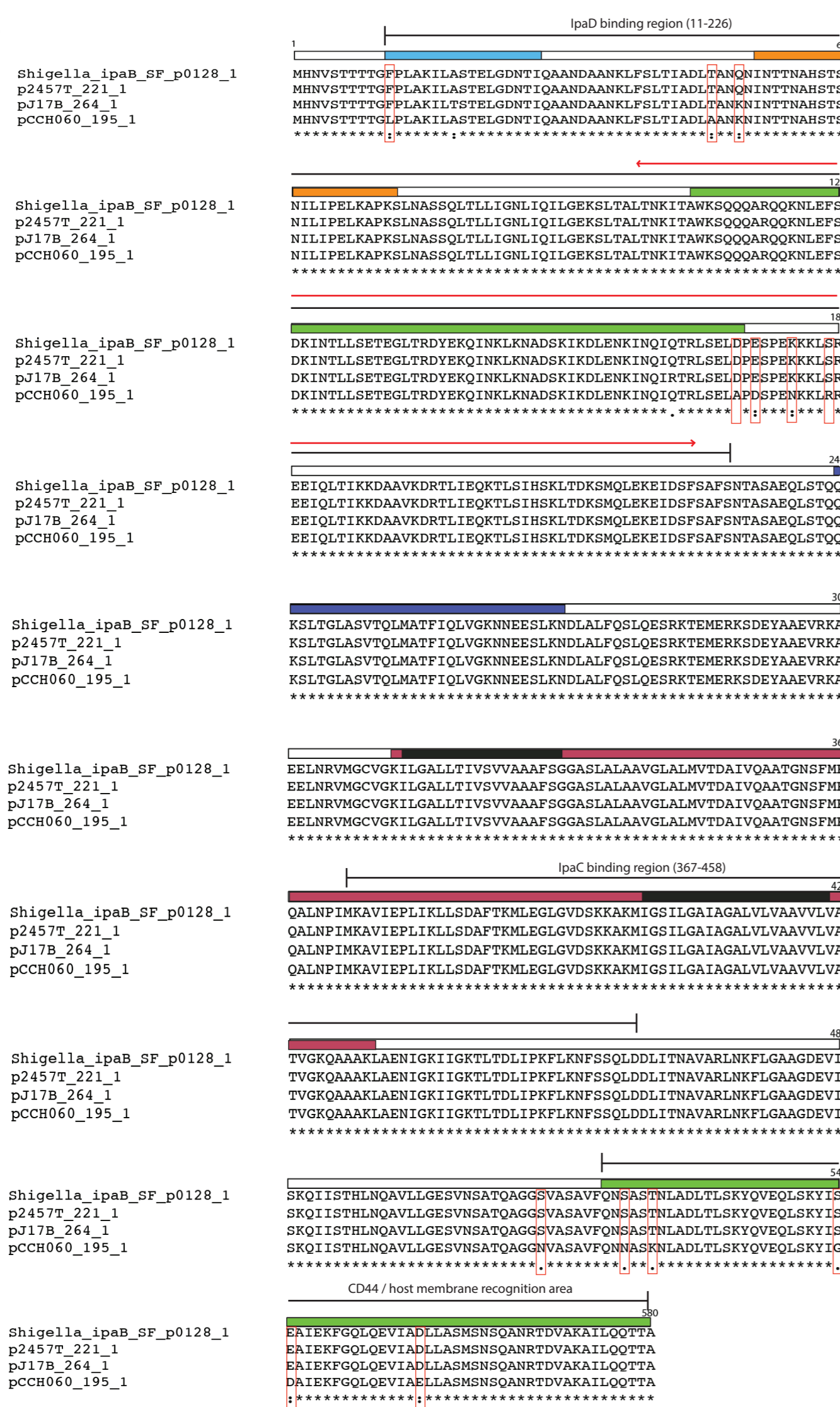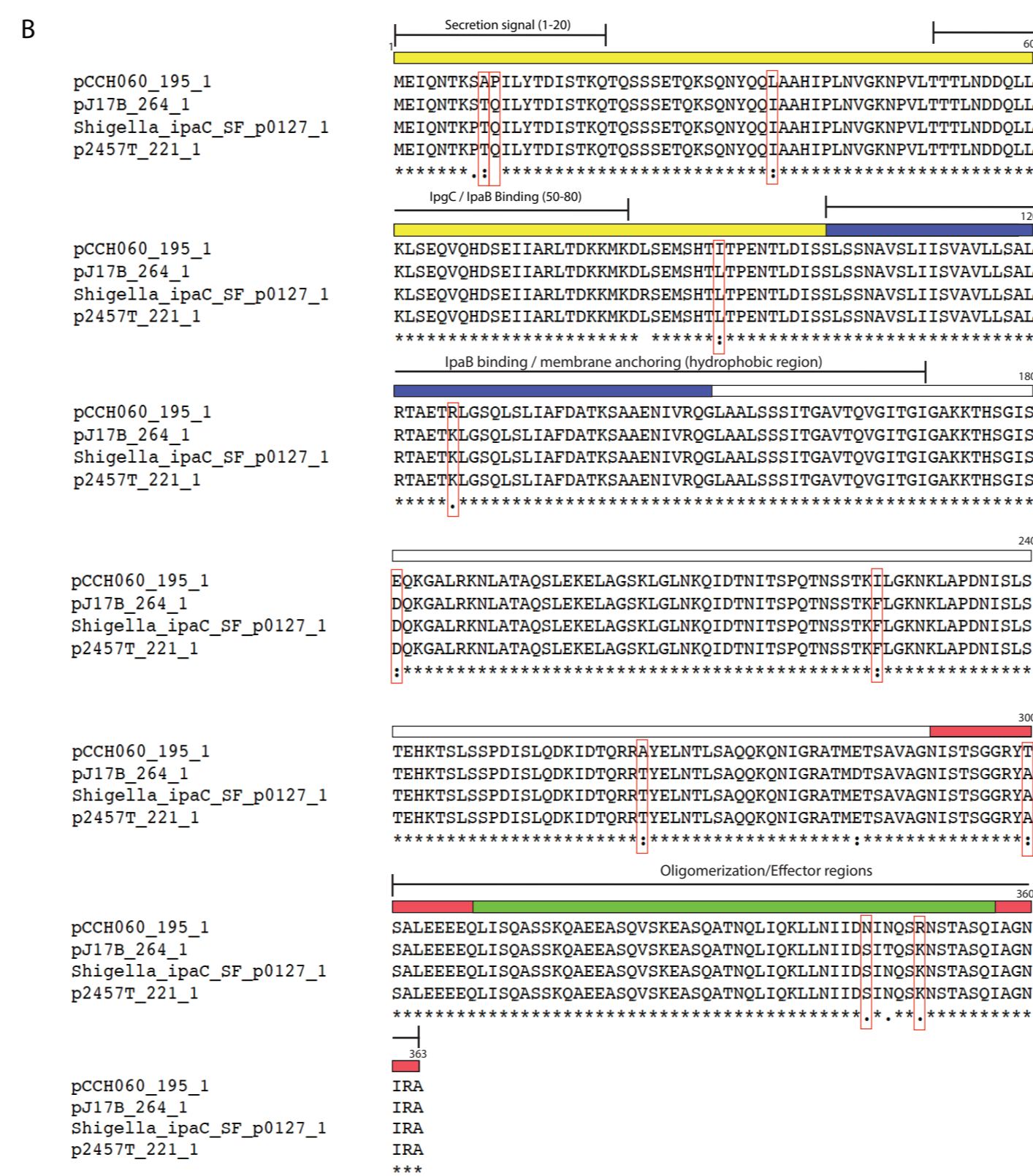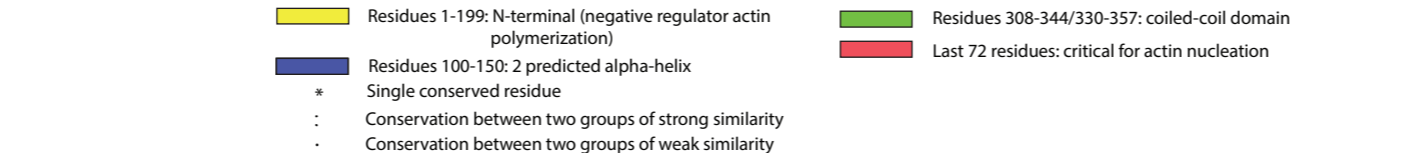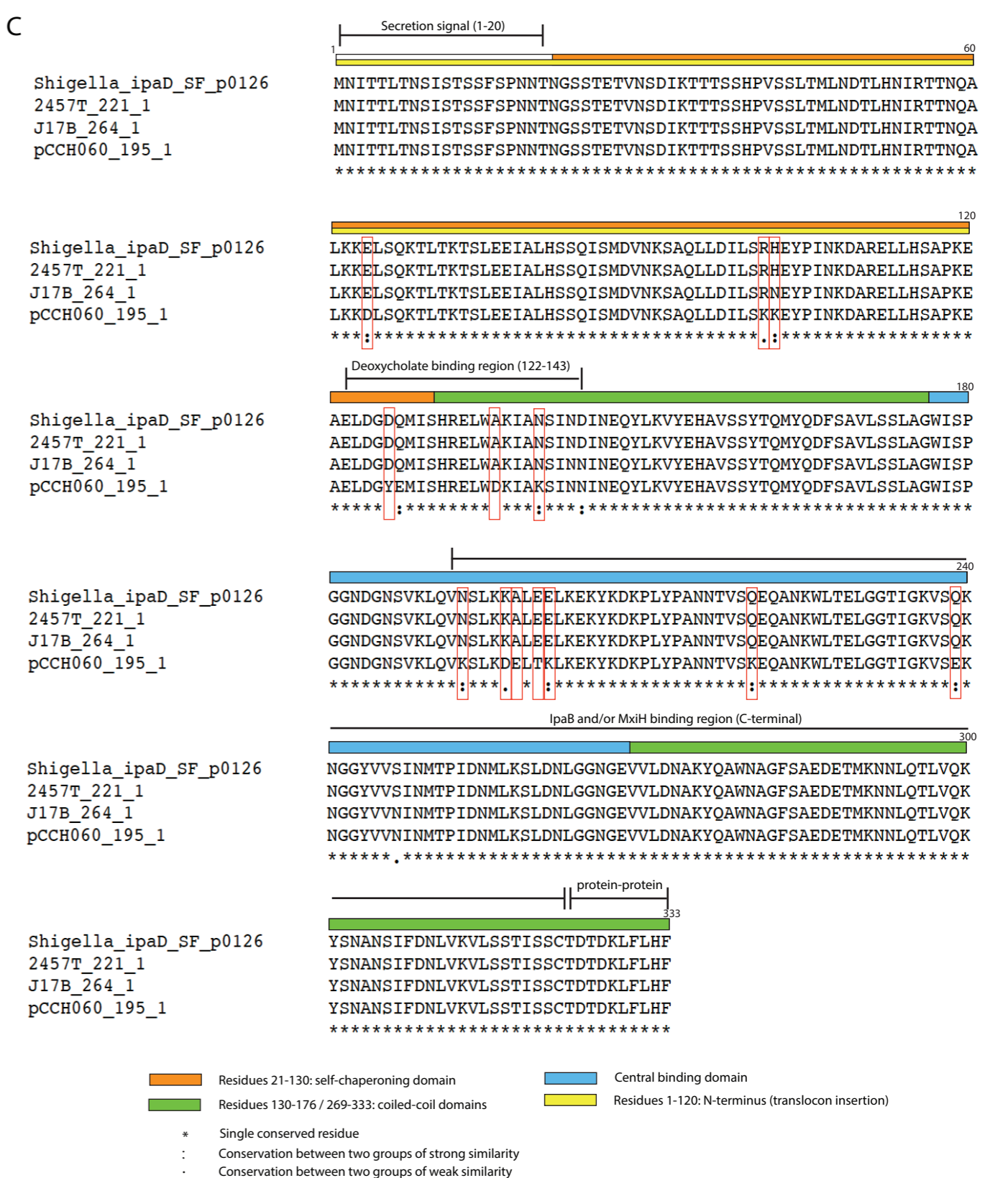

Supplement: Figure S4 — Amino acid alignments. [file msphere.00408-23-s0007.pdf]

A

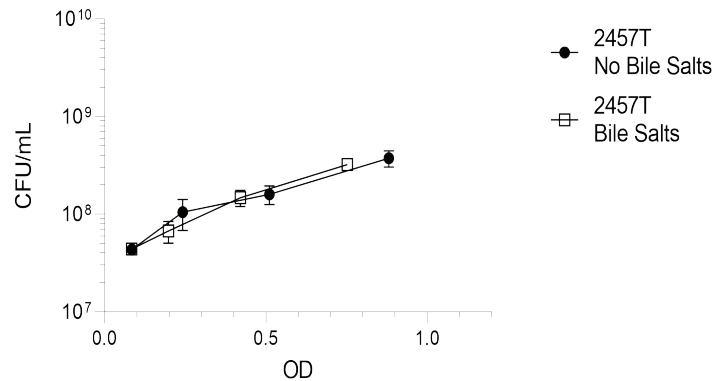

B

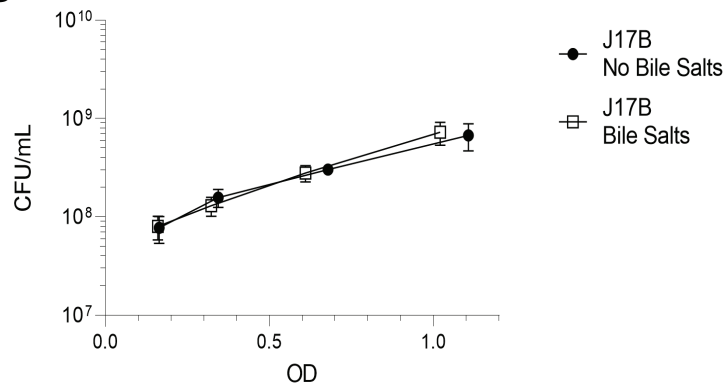

C

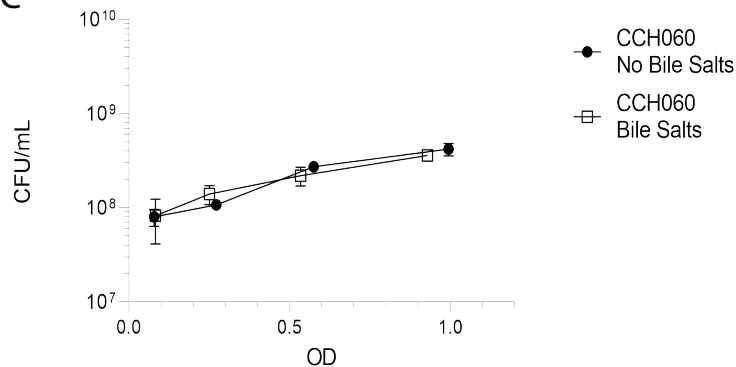

Supplement: Figure S5 — Growth curve. [file msphere.00408-23-s0008.pdf]
