# Supplementary material for: Genomic, transcriptomic, and phenotypic differences among archetype Shigella flexneri strains of serotypes 2a, 3a, and 6
Source: mSphere. 2023 Oct 13;8(6):e00408-23. doi: 10.1128/msphere.00408-23 (PMC10732043; doi:10.1128/msphere.00408-23)
Supplement: Legends — Supplemental legends. [file msphere.00408-23-s0009.docx]

Supplemental Material for:

Genomic, transcriptomic, and phenotypic differences among archetype *Shigella flexneri* strains of serotypes 2a, 3a, and 6

Caitlin E. Gabor^1,2,4^, Tracy H. Hazen^1,2^, BreOnna C. Delaine-Elias^4^

David A. Rasko^1,2,3^ and *Eileen M. Barry^2,4^

^1^Institute for Genome Sciences, University of Maryland School of Medicine, Baltimore, MD, USA

^2^Department of Microbiology and Immunology, University of Maryland School of Medicine, Baltimore, MD, USA

^3^Center for Pathogen Research, University of Maryland School of Medicine, Baltimore, MD 21201.

^4^Center for Vaccine Development and Global Health, University of Maryland School of Medicine, Baltimore, MD, USA

*Corresponding author:

Eileen M. Barry : embarry@som.umaryland.edu

**Supplemental Materials**

Table S1. Strains included in phylogenomic analyses

Table S2. Virulence plasmid cross comparison LS-BSR

Table S3. RNA-seq mapping summary

Table S4. PCR/qRT-PCR Primers

Figure S1. Invasion with increased CCH060 inoculum

Figure S2. Total protein and DnaK expression

Figure S3. Quantification of *ipaB, ipaC,* and *ipaD* transcription using qRT-PCR on bacteria grown in bile salt and non-bile salt conditions.

Figure S4. Predicted amino acid sequence alignment for IpaB, IpaC, and IpaD

Figure S5. Growth curves for archetype strains

Dataset 1. Annotation dataset

Dataset 2. LS-BSR matrices

Dataset 3. RNA-seq complete matrix file

**Figure S1. Invasion with increased CCH060 inoculum.** *S. flexneri* archetype isolates infected HT-29 monolayers for 90min. *S. flexneri* isolate 2457T was inoculated at MOI 1:100 while CCH060 was inoculated at an MOI of 1:100, 1:400, 1:800. Cells were lysed to enumerate intracellular bacteria at 2h pi by CFU/ml (**panel A**) or normalized by inoculum to calculate percent recovery (**panel B**). The asterisks above indicate statistically significant differences determined using an ANOVA and Tukey posttest; ***, P<0.0001; ****, P<0.0001.

**Figure S2. Supernatant and whole cell lysate protein analysis in bile salt conditions.** Supernatant and corresponding whole cell lysates were collected during TSB and TSB+deoxycholate growth conditions (Figure 6A, 6B). Total protein was analyzed for supernatant **(panel A)** and corresponding whole cell lysates **(panel B)** using GelCode. Each archetype strain and growth condition were collected at mid-log phase (OD_600_ ~0.7; 2x10^8^ CFU/ml). Total protein analyses was performed to confirm equal loading of samples (Figure 7B) and bile salt effect was in accordance with previous literature (Figure 7)(59). DnaK **(panel C)** was used as a loading control to normalize whole cell lysates to calculate Ipa protein levels using densitometry (Fiji ImageJ).

**Figure S3. Quantification of *ipaB, ipaC,* and *ipaD* transcription using qRT-PCR on bacteria grown in bile salt and non-bile salt conditions.** Whole cell lysates from corresponding western blot samples collected from the TSB or TSB+deoxycholate growth conditions described previously (Figure 7 and Figure S3) were analyzed for *ipaB* **(panel A)**, *ipaC* **(panel B)**, and *ipaD* **(panel C)** transcription rate. Collections and analyses were performed in triplicate (n=3). Primers set used can be found in Table S4. The asterisks above indicate statistically significant differences determined using an ANOVA and Tukey posttest; *, P<0.05; **, P<0.01; ****, P<0.0001.

**Figure S4. Predicted amino acid sequence alignment for IpaB, IpaC, and IpaD.** Nucleotide sequences for IpaBCD proteins were obtained from each archetype strain’s pINV complete sequence and aligned to *S. flexneri* strain 301T’s *ipaBCD* (data not shown) using MAFFT FFT-NS-I v7.487 (96). Nucleotide sequences were translated using EMBOSS Transeq (97) and aligned with Clustal MUSCLE v3.8 (98). IpaB (**panel A**), IpaC (**panel B**), and IpaD (**panel C**) functional domains are indicated above the sequence based on previous literature (37, 66, 67, 99-101). Red boxes indicate locations where amino-acid substitutions are present uniquely for CCH060.

**Figure S5. Archetype strain growth curves.** *S. flexneri* archetype strains 2457T (panel A), J17B (panel B), and CCH060 (panel C) were grown for 2 hours in TSB or TSB+0.1% deoxycholate. The OD_600_ and enumeration for CFU/ml was recorded every 30min.
