# Supplementary material for: Genomic, transcriptomic, and phenotypic differences among archetype Shigella flexneri strains of serotypes 2a, 3a, and 6
Source: mSphere. 2023 Oct 13;8(6):e00408-23. doi: 10.1128/msphere.00408-23 (PMC10732043; doi:10.1128/msphere.00408-23)
Supplement: Supplemental Tables — Tables S1-S4. [file msphere.00408-23-s0010.docx]

| **Table S1.** Strains included in phylogenomic analyses | | | |  |
| --- | --- | --- | --- | --- |
| Strain Designation | Serotype | *S. flexneri* PG | Accession/Sequencing ID | |
| **CCH060** | Sf6 | none | CP099865, CP099866, CP09867 | |
| 6-645500 | Sf6 | none | GCA_002949515.1 | |
| 700918 | Sf6 | none | ERR6005728 | |
| 603694 | Sf6 | none | ERR6005544 | |
| 500067 | Sf6 | none | ERR6004942 | |
| 400603 | Sf6 | none | ERR6004796 | |
| 204161 | Sf6 | none | ERR6004748 | |
| 300039 | Sf6 | none | ERR6004758 | |
| 102609 | Sf6 | none | ERR6004643 | |
| 5.63 | 1a | PG1 | ERS093677 | |
| 262.78 | 1a | PG1 | ERS093700 | |
| Sh07-3008 | 1a | PG1 | ERS025936 | |
| 439 | 1a | PG3 | GCA_0022401225.1 | |
| 670 | *1a* | PG3 | GCA_002240135.1 | |
| 509/60 | 1b | PG1 | ERS088061 | |
| K-10302 | 1b | PG1 | ERS157622 | |
| KP-15 | 1b | PG1 | ERS157813 | |
| Sh08-2354 | 1b | PG1 | ERS025947 | |
| K-2535 | 1c | PG1 | ERS157767 | |
| K-9590 | 1c | PG1 | ERS157816 | |
| Sh09-1985 | 1c | PG1 | ERS025954 | |
| MS-341 | 1cv | PG1 | ERS157773 | |
| 3.59 | 2a | PG3 | ERS093675 | |
| 410370 | 2a | PG3 | ERS087989 | |
| G1663 | 2a | PG3 | GCA_001021855.1 | |
| 301 | 2a | PG3 | GCA_000006925.2 | |
| ATCC 29903 | 2a | PG3 | GCA_002950215.1 | |
| **2457T** | 2a | PG3 | CP100044, CP100045, CP100046, CP100047, CP100048 | |
| IB0712 | 2a | PG3 | ERS033355 | |
| 981 | 2a | PG3 | GCA_001580175.1 | |
| 179-89 | 2b | PG1 | ERS093703 | |
| Sh09-1522 | 2b | PG1 | ERS025953 | |
| K-373 | 2b | PG3 | ERS157663 | |
| Sh04-2878 | 2b | PG3 | ERS025907 | |
| 428777 | 3a | PG2 | ERS088021 | |
| K-10248 | 3a | PG2 | ERS157670 | |
| K-734 | 3a | PG2 | ERS157674 | |
| Sh09-4409 | 3a | PG2 | ERS025956 | |
| NCDC 2825-H | 3a | PG4 | ERS093705 | |
| Sh08-0350 | 3a | PG4 | ERS025944 | |
| **J17B** | 3a | PG4 | CP100042, CP100043 | |
| IB4229 | 4a | PG7 | ERS033385 | |
| Sh06-1976 | 4a | PG7 | ERS025928 | |
| K-9108 | 4av | PG1 | ERS157823 | |
| Sh05-1382 | 4av | PG1 | ERS025913 | |
| IB2494 | 4av | PG7 | ERS033364 | |
| 34/53 | 4b | PG1 | ERS088069 | |
| NCDC 1862-71 | 4b | PG1 | ERS093692 | |
| CIP 52-24 | 4bv | PG4 | ERS093707 | |
| IB1651 | 4bv | PG4 | ERS033311 | |
| 1602 | 4c | PG3 | GCA_002240075.1 | |
| 1205 | 4c | PG3 | GCA_001579965.1 | |
| NCDC 1170-74 | 5a | PG3 | ERS093697 | |
| 192/51 | 5a | PG5 | ERS088071 | |
| 74-1170 | 5a | PG3 | GCA_002950255.1 | |
| CIP 67-61 | 5a | PG5 | ERS093681 | |
| 13-55 | 5b | PG5 | ERS088070 | |
| IB1665 | 5b | PG5 | ERS033325 | |
| NCTC 9728 | 5b | PG5 | GCA_002950335.1 | |
| 94-3007 | 7b | PG1 | GCA_002741635.1 | |
| 2002017 | Fxv | PG3 | GCA_000022245.1 | |
| FDAARGOS_74 | N/A | PG2 | GCA_000783835.2 | |
| 89-141 | N/A | PG6 | GCS_002946695.1 | |
| NCDC 9768 | X | PG1 | ERS093711 | |
| IB0036 | X | PG3 | ERS033372 | |
| Sh07-5876 | X | PG3 | ERS025940 | |
| 531/61 | X | PG4 | ERS088074 | |
| IB1773 | X | PG7 | ERS033384 | |
| Sh08-1372 | Xv | PG3 | ERS025946 | |
| Sh09-6999 | Xv | PG3 | ERS025962 | |
| Sh06-8628 | Y | PG1 | ERS025932 | |
| IB1757 | Y | PG3 | ERS033382 | |
| Sh05-4557 | Y | PG3 | ERS025917 | |
| K-9165 | Y | PG6 | ERS157764 | |
| MS-228(a) | Y | PG6 | ERS157757 | |
| IB0017 | Y | PG7 | ERS033369 | |
| IB1709 | Y | PG7 | ERS033360 | |
| 93-3063 | Y | PG1 | GCA_002949575.1 | |
| IVI-77/AR3444 | Yv | PG6 | ERS157774 | |
| K-9144(b) | Yv | PG6 | ERS157772 | |
| IB1716 | Yv | PG7 | ERS033380 | |
| Sh07-5519 | Yv | PG7 | ERS025939 | |
|  |  |  |  | |
| Strain Designation | Description | Phylogroup | Accession No. | |
| BL21 | lab adapted | A | NC_012947.1 | |
| ATCC 8739 | lab adapted | A | NC_010468.1 | |
| 53638 | EIEC | A | AAKB00000000.2 | |
| H10407 | ETEC | A | GCA_000210475.1 | |
| K12 str. MG1655 | commensal | A | NC_000913.3 | |
| HS | commensal | A | NC_009800.1 | |
| IAI1 | fecal isolate | B1 | NC_011741.1 | |
| 55989 | EAEC | B1 | NC_011748.1 | |
| 11368 | EHEC | B1 | NC_013361.1 | |
| 11128 | EHEC | B1 | NC_013364.1 | |
| B171 | tEPEC | B1 | AAJX00000000.2 | |
| E110019 | aEPEC | B1 | AAJW00000000.2 | |
| E24377A | ETEC | B1 | NC_009801.1 | |
| B7A | ETEC | B1 | AAJT00000000.2 | |
| C227-11 | STEC/EAEC | B1 | GCA_000986765.1 | |
| *S. boydii* 3083-94 | *Shigella* | B1 | GCA_000020185.1 | |
| *S. sonnei* 046 | *Shigella* | B1 | GCA_000092525.1 | |
| SE11 | commensal | B1 | GCA_000010385.1 | |
| 536 | UPEC/ExPEC | B2 | NC_008253.1 | |
| S88 | ExPEC/NMEC? | B2 | NC_011742.1 | |
| UTI89 | UPEC/ExPEC | B2 | NC_007946.1 | |
| CFT073 | UPEC/ExPEC | B2 | AE014075.1 | |
| E2348/69 | tEPEC | B2 | NC_011601.1 | |
| ED1a | commensal | B2 | CU928162.2 | |
| APEC O1 | APEC | B2 | GCF_000014855.1 | |
| APEC 789 | APEC | C | CP010315 | |
| APEC 078 | APEC | C | CP004009 | |
| UMN026 | UPEC/ExPEC | D | NC_011751.1 | |
| 042 | EAEC | D | GCA_000027125.1 | |
| EDL933 | EHEC | E | GCA_000006665.1 | |
| Sakai | EHEC | E | GCA_000008865.1 | |
| CB9615 | EPEC | E | NC_013941.1 | |
| *S. dysenteriae* 197 | *Shigella* | E | GCA_000012005.1 | |
| SMS_3_5 | environmental | F | CP000970.1 | |
| IAI39 | UPEC/ExPEC | F | NC_011750.1 | |
| 200177 | commensal | G | JAAPEA000000000 | |
| 700491 | commensal | G | JAASSXC000000000 | |

| **Table S2.** Archetype virulence plasmid comparison | | | | |  | |  | |  | |  | |  | | |
| --- | --- | --- | --- | --- | --- | --- | --- | --- | --- | --- | --- | --- | --- | --- | --- |
| Reference | Total genes in plasmid | Shared between 3 | Shared between 2 | | | | | Unique | | | | | | |  |
|  |  |  | 2457T + J17B | 2457T + CCH060 | | CCH060 + J17B | | 2457T | | J17B | | CCH060 | |  |  |
| 2457T_p221 | 268 | 80.5% (216/268) | 10.8% (29/268) | 2.6% (7/268) | | - | | 4.8% (13/268) | | - | | - | |  |  |
| J17B_p264 | 368 | 81.5% (300/368) | 9.8% (36/368) | - | | 0.5% (2/368) | | - | | 4.3% (16/368) | | - | |  |  |
| CCH060_p195 | 247 | 83.4% (206/247) | - | 0.4% (1/247) | | 1.2% (3/247) | | - | | - | | 9.7% (24/247) | |  |  |
| Average Total |  | **81.80%** | **10.30%** | **1.50%** | | **6.25%** | | **4.80%** | | **4.30%** | | **9.70%** | |  |  |

| **Table S3.** RNA-seq mapping summary | | | | |  | |  | |  | |  | |  | |  | |
| --- | --- | --- | --- | --- | --- | --- | --- | --- | --- | --- | --- | --- | --- | --- | --- | --- |
| Isolate | Serotype | Condition | Replicate | Total Reads | | Total Reads Mapped | | % of Reads Mapped | | % of Reads Mapped to Genes | | % of Reads Mapped to Intergenic Regions | | SRA Accession No. | |  |
| 2457T | 2a | control | 1 | 64,382,914 | | 24,631,148 | | 38.26 | | 78.48 | | 21.52 | | SRR19886883 | |  |
|  |  | control | 2 | 103,981,000 | | 39,325,916 | | 37.82 | | 81.36 | | 18.64 | | SRR19886882 | |  |
|  |  | bile | 1 | 75,937,708 | | 22,836,700 | | 30.07 | | 76.29 | | 23.71 | | SRR19886879 | |  |
|  |  | bile | 2 | 106,181,892 | | 34,331,188 | | 32.33 | | 70.5 | | 29.5 | | SRR19886878 | |  |
| J17B | 3a | control | 1 | 82,178,970 | | 21,518,194 | | 26.18 | | 75.65 | | 24.35 | | SRR19886877 | |  |
|  |  | control | 2 | 90,005,530 | | 18,468,080 | | 20.52 | | 67.73 | | 32.27 | | SRR19886876 | |  |
|  |  | bile | 1 | 70,985,736 | | 25,034,204 | | 35.27 | | 64.43 | | 35.57 | | SRR19886875 | |  |
|  |  | bile | 2 | 77,832,202 | | 15,833,430 | | 20.34 | | 66.81 | | 33.19 | | SRR19886874 | |  |
| CCH060 | 6 | control | 1 | 93,692,308 | | 36,405,368 | | 38.86 | | 79.84 | | 20.16 | | SRR19886873 | |  |
|  |  | control | 2 | 98,397,992 | | 42,189,356 | | 42.88 | | 82.54 | | 17.46 | | SRR19886872 | |  |
|  |  | bile | 1 | 103,995,984 | | 31,604,796 | | 30.39 | | 78.57 | | 21.43 | | SRR19886881 | |  |
|  |  | bile | 2 | 108,468,518 | | 36,595,904 | | 33.74 | | 77.90 | | 22.10 | | SRR19886880 | |  |

| **Table S4.** PCR and qRT-PCR Primers | | |  | |
| --- | --- | --- | --- | --- |
| **PCR Primers** | | | |  |
|  | Name | 5' - 3' Sequence | Reference | |
| Primer Set 1 | OspG F | GGG TCG ACA GCA GAA ATC TT | NC_004851.1:183824-184414 Shigella flexneri 2a str. 301 plasmid pCP301 | |
|  | OspG R | GTT GCA GCG TAA TAT TCA GCA TAA |  |  |
| Primer Set 2 | SepA F | CGG GAG ACC ATA CAC TTT ACT C | NC_004851.1:c56839-52745 Shigella flexneri 2a str. 301 plasmid pCP301 | |
|  | SepA R | GTG GTG ACG ACC CAG TTA TT |  |  |
| Primer Set 3 | ShiA F | GCG GCA GGT AAT CGT ATC AT | NC_004337.2:3808392-3809435 Shigella flexneri 2a str. 301 chromosome | |
|  | ShiA R | CAG ATT TCC GCA CAG CAT TTC |  |  |
| Primer Set 4 | Pic F | CTT GTG GGA AGA CTG GGT AAA G | NC_004337.2:c3071855-3067737 Shigella flexneri 2a str. 301 chromosome | |
|  | Pic R | TAC CAC TAC CCA CCC GAT AAA |  |  |
| Primer Set 5 | SigA F | TGG ACC CTG ACC GGA TAT AA | NC_004337.2:3060437-3064294 Shigella flexneri 2a str. 301 chromosome | |
|  | SigA R | CTG TGA CTT TCC AGT CCT TAC |  |  |
| Primer Set 6 | gtrII F | TGA TAC TCT GCG AAG ACA GAA AG | NC_004337.2:319152-320612 Shigella flexneri 2a str. 301 chromosome | |
|  | grtII R | GAT TCG AGG AAG GTA AA |  |  |
| **qRT-PCR Primers** | | | |  |
|  | Name | 5' - 3' Sequence | Reference | |
| Primer Set 1 | IpaB F | TCT GGC TCT ATT CCA GTC TCT C | NZ_CP037924.1: 196776-198518 Shigella flexneri 5a str. M90T plasmid CP037924.1 | |
|  | IpaB R | TCC CAA CAC AAC CCA TTA CTC |  |  |
| Primer Set 2 | IpaC F | GTT CTA CTT TCT GCT CTC CG | NZ_CP037924.1: 198481-199629 Shigella flexneri 5a str. M90T plasmid CP037924.1 | |
|  | IpaC R | TGA TTT TGT AGC ATC GAA CGC |  |  |
| Primer Set 3 | IpaD F | CCA CCT CAT CAT TCA GTC CAA | NZ_CP037924.1: 199680-200678 Shigella flexneri 5a str. M90T plasmid CP037924.1 | |
|  | IpaD R | AAG GGT GTC GTT GAG CAT AG |  |  |
| Primer Set 4 | RpoA F | TGT AGG CAA TAC GCT CCA CA | Hazen et al. 2017 [87] | |
|  | RpoA R | GGT TAT GTG CCG GCT TCT AC |  |  |
